# Structure Elucidation of Calyxoside B, a Bipolar Sphingolipid from a Marine Sponge *Cladocroce* sp. through the Use of Beckmann Rearrangement

**DOI:** 10.3390/md19060287

**Published:** 2021-05-21

**Authors:** Kenji Sugawara, Hiroshi Watarai, Yuji Ise, Hisayoshi Yokose, Yasuhiro Morii, Nobuhiro Yamawaki, Shigeru Okada, Shigeki Matsunaga

**Affiliations:** 1 Laboratory of Aquatic Natural Products Chemistry, Graduate School of Agricultural and Life Sciences, The University of Tokyo, 1-1-1 Yayoi, Bunkyo-ku, Tokyo 113-8657, Japan; sugawara-kenji525@g.ecc.u-tokyo.ac.jp (K.S.); aokada@mail.ecc.u-tokyo.ac.jp (S.O.); 2 Department of Immunology and Stem Cell Biology, Faculty of Medicine, Kanazawa University, Ishikawa 920-8640, Japan; hwatarai@med.kanazawa-u.ac.jp; 3 Sesoko Station, Tropical Biosphere Research Center, University of the Ryukyus, 3422 Sesoko, Motobu, Okinawa 905-0227, Japan; h206944@lab.u-ryukyu.ac.jp; 4 Graduate School of Science and Technology, Kumamoto University, 2-39-1 Kurokami, Chuo-ku, Kumamoto 860-8555, Japan; yokose@kumamoto-u.ac.jp; 5 Graduate School of Fisheries Science and Environmental Studies, Nagasaki University, Nagasaki 852-8521, Japan; yasu-m@nagasaki-u.ac.jp (Y.M.); yamawaki@nagasaki-u.ac.jp (N.Y.)

**Keywords:** marine natural products, marine sponge, glycosphingolipid, structure elucidation

## Abstract

Marine sponges are an excellent source of biologically active secondary metabolites. We focus on deep-sea sponges for our discovery study. A marine sponge *Cladocroce* sp. exhibited cytotoxic activity in the bioactivity screening. From this sponge a previously unreported cytotoxic glycosphingolipid, calyxoside B, was isolated and the structure of this compound was elucidated by analyses of MS and NMR spectra and chemical derivatization. We converted the ketone in the middle of a long aliphatic chain into an oxime to which was applied Beckmann rearrangement to afford two positional isomers of amides. The products were subjected to acidic hydrolysis followed by LC-MS analysis, permitting us to assign unequivocally the position of the ketone. Calyxoside B shows cytotoxicity against HeLa cells with an IC_50_ value of 31 µM and also weakly stimulated the production of cytokines in mice.

## 1. Introduction

From marine sponges a wide variety of bioactive secondary metabolites with unique structures have been discovered [1]. However, due to a great deal of effort devoted by natural product chemists to study their constituents, the frequency of rediscovery of known compounds has increased in recent years. Because of the lesser accessibility of deep-sea sponges, they are generally underexplored compared with those from shallow waters [2]. Investigation of such an untapped resource would lead to the discovery of new biologically active secondary metabolites. In our continuing cytotoxic screening of such resources [3,4], the extract of a marine sponge, *Cladocroce* sp., exhibited activity against HeLa human cancer cervical cells. We have isolated calyxoside B (**1**) together with the known compound calyxoside (**2**) [5]. We have determined the structure of **1** by interpretation of spectroscopic data combined with the modified Mosher’s analysis of an appropriately protected derivative and conversion of the amino alcohols into oxazolidinones. The position of the ketone in the middle of the long alkyl chain was determined after converting the ketone into two positional isomers of amides by the Beckmann rearrangement.

## 2. Results

### 2.1. Isolation of Cytotoxic Constituents

**Figure 1:**
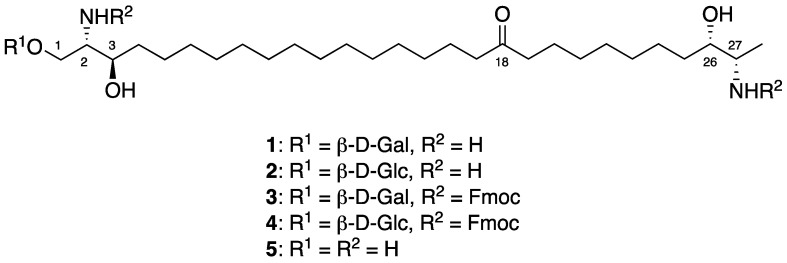
Structures of **1**–**5**.

The combined MeOH and CHCl_3_-MeOH (1:1) extract of the marine sponge was subject to solvent portioning, ODS flash column chromatography, and reversed phase HPLC to give a 1:3 mixture of **1** and **2**. It was not possible for us to separate the two compounds as the natural forms. Therefore, we derivatized the amino group of each compound with the Fmoc group to afford a mixture of **3** and **4** (Figure 1), which were separated by recycle HPLC with 20 cycles. After the removal of the Fmoc group from **3** and **4**, calyxoside B (**1**) and calyxoside (**2**) were obtained. Although **2** was isolated in high purity, **1** still contained ca. 20% of **2** as judged from the 1H and 13C NMR spectra. Compounds **1** and **2** were isomeric to the known compound calyxoside isolated from a marine sponge *Calyx* sp. Although ^1^H and ^13^C NMR data of **2** were very similar to those of calyxoside, it was not possible to conclude their identity by comparing their spectroscopic data, because two chiral head groups were separated by a long alkyl chain and the location of the carbonyl group could not be assigned from the NMR data.

### 2.2. Structure Elucidation

**Figure 2:**
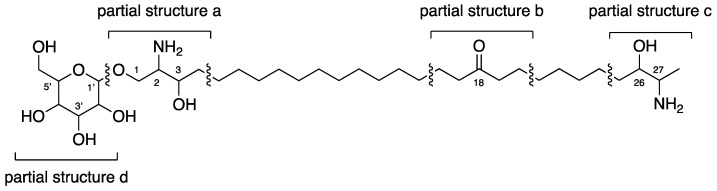
Partial structures of **1**.

The molecular formula of calyxoside B (**1**) was determined as C_34_H_69_N_2_O_9_ by HRESIMS (*m/z* 649.5003 [M + H]^+^). The ^1^H and ^13^C NMR data shows the presence of one methyl, many unfunctionalized methylenes, two oxygenated methylenes, five oxygen-bearing and two nitrogen-bearing methines, one acetal, and one ketone (Table 1). Interpretation of the COSY and HSQC data permitted us to establish partial structures a-d (Figure 2). The partial structure constituted one terminus of an aliphatic chain in which C-1 and C-3 were substituted by an oxygen atom and C-2 by a nitrogen atom judging from the ^1^H and ^13^C NMR chemical shifts (Table 1). Partial structure b was a ketone substituted by two linear alkyl groups. Partial structure c was composed of a terminal methyl attached to a nitrogen-bearing methine, which was connected to an oxygenated methine. Partial structure d contained the remaining six oxygenated carbons including one acetal, four oxygenated methines, and one oxygenated methylene, suggesting the presence of a hexose. ^1^H NMR data, specifically the magnitudes of ^1^H-^1^H coupling constants within this system permitted us to assign this unit as a β-galactopyranosyl unit, which was supported by the ^13^C NMR data (Table 1). The observation of a HMBC correlation between H-1′ and C-1 revealed that the galactose moiety is connected to C-1. Because the remaining carbons were all aliphatic methylenes, partial structures a and b, as well as partial structures b and c, were connected through unbranched alkyl chains. It was not possible to determine the length of each chain due to overlapped methylene signals in the ^1^H and ^13^C NMR spectra (Figures S2 and S4). Compound **2** contained the same partial structures except for partial structure d, which was glucopyranosyl.

**Scheme 1:**
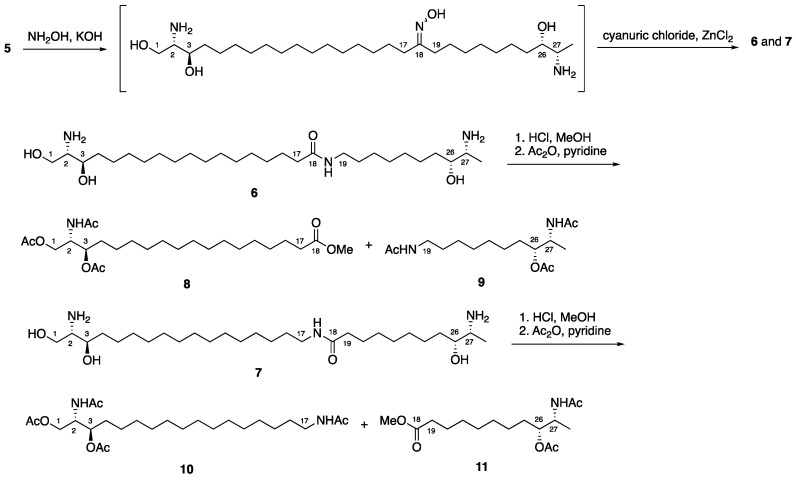
Acidic hydrolysis products of **6** and **7**, which were produced by the Beckmann rearrangement of **5**.

The position of the ketone was studied by the hydrolysate of the mixture of **1** and **2**, which gave the homogeneous aglycone **5** as judged from the NMR data and confirmed by the degradation study described below (Figure S12). The position of the carbonyl carbon in this class of compounds was reported to be difficult to determine by mass spectrometry [6]. We anticipated that the conversion of the ketone into amide would permit the assignment of the position of the ketone due to the facile cleavage of the amide bond by acid. Because a mild reaction to convert ketoximes to amides has been reported, we elected to use this reaction for our purpose [7]. Compound **5** was converted to the oxime which was subjected to the Beckmann rearrangement to afford a mixture of amides **6** and **7** (Scheme 1). This mixture was subjected to methanolysis followed by acetylation and LC-MS analysis. Four peaks with protonated molecules at *m/z* 315.22, 316.20, 471.34, and 472.32 were detected, demonstrating that **8**–**11** were formed (Figure S14). This result showed that **5** derived from the mixture of **1** and **2** was homogeneous and the ketone was placed at C-18 [5]. In order to confirm this result, we isolated **6** and analyzed it in the same way, which gave LC-MS peaks corresponding to **8** and **9**. This experiment confirmed the position of the ketone as C-18 in both **1** and **2**.

**Figure 3:**
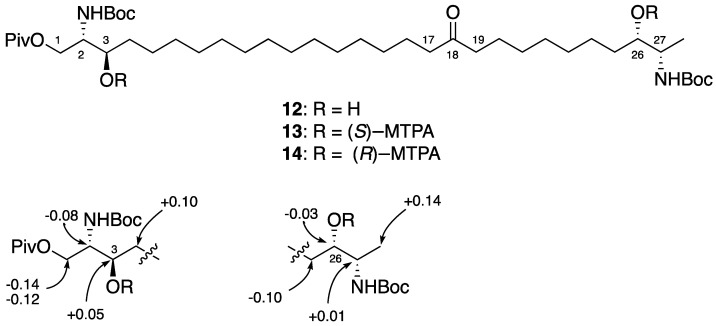
Structures of **12**–**14** and Δδ values for the MTPA esters of **12**.

We then studied the absolute configuration of the four stereogenic centers in **5**. Although the absolute configuration of the aglycone of calyxoside had already been established [5], it was not possible to discuss the identity or difference between the two aglycones, because the NMR data of the aglycone of calyxoside was not reported and the two head groups were insulated by a long alkyl chain. Therefore, we sought to assign the absolute configuration of the aglycone **5** independently. We first studied the absolute configurations of the secondary alcohols at C-3 and C-26. Each amino group in **5** was protected with a Boc group, and the primary alcohol at C-1 was protected with the pivaloyl group to give **12**, which was subjected to the modified Mosher’s analysis by converting to the MTPA esters, **13** and **14** [8] (Figure 3). The distribution of the Δδ values showed that the configuration of C-3 was *R*, because the Δδ values of H_2_-1 and H-2 were negative and those of H_2_-4 were positive. C-26-*S* configuration was assigned, because the Δδ values of H_2_-25 were negative and those of H_3_-28 were positive.

**Scheme 2:**
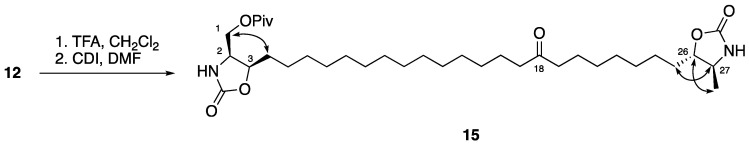
Preparation of the cyclic carbamates **15**. Key NOESY correlations are shown by arrows.

We examined the relative configuration of the vicinal amino alcohol in each head group after conversion to an oxazolidinone. The cis- and trans-disubstituted oxazolidinones were reported to be distinguishable by the NOESY data, though the values of vicinal coupling constants were almost identical between the two isomers [9,10]. Deprotection of the Boc groups in **12** followed by treatment with *N*, *N*’-carbonyldiimidazole (CDI) gave the bis-oxazolidinone **15** (Scheme 2). In the oxazolidinone ring including C-2 and C-3, a conspicuous NOESY correlation was observed between H-1a and H_2_-4, suggesting that H-2 and H-3 were cis. In the oxazolidinone ring including C-26 and C-27, NOESY cross peaks were observed between H-26 and H_3_-28 and between H_2_-25 and H-27, indicating that H-26 and H-27 were trans (Scheme 2). With the absolute configurations of C-3 and C-26 already determined, the absolute configuration of **5** was established as 2*S*, 3*R*, 26*S,* 27*S*, which is identical to the proposed absolute configuration of calyxoside [5].

The absolute configurations of the sugar moiety were studied by using a mixture of **1** and **2**. The mixture was hydrolyzed and the liberated hexoses were converted into 3-phenylthiocarbamoylthiazoline-4(*R*)-carboxylates [11]. The LC-MS analysis showed the presence of D-glucose and D-galactose (Figure S24), demonstrating that the galactose and glucose in **1** and **2**, respectively, were both in the D-form. Therefore, **2** was calyxoside itself and **1** was its galactosyl form.

### 2.3. Biological Activity

Calyxoside B (**1**) and calyxoside (**2**) show moderate cytotoxicity against HeLa cells with IC_50_ values of 31 and 12 µM, respectively. Compound **5** and the mixture of **6** and **7** also exhibited cytotoxicity with IC_50_ values of 2.5 and 21 µM, respectively. It is interesting to note that the aglycone showed the most potent activity and conversion of the ketone in the aglycone to an amide resulted in diminished activity. Calyxosides are distantly related to agelasphins—sponge-derived sphingolipids—by possessing 1-glycosylated sphingolipid partial structures [12]. Agelasphins and their synthetic analogue KRN7000 are presented by MHC class I-like antigens presenting molecule CD1d and exhibit antitumor activity through enhanced production of cytokines by direct stimulation of invariant natural killer T cells (iNKT cells) [12]. Therefore, we wondered whether a mixture of **1** and **2** exhibits similar activities. We have examined the production of interferon-γ, interleukin-13, and interleukin-17A from spleen cells of C57BL/6 wild-type mice or of *Cd1d*-deficient mice upon treatment with KRN7000 and a 3:1 mixture of **1** and **2**. *Cd1d*-deficient mice lack iNKT cells and cannot produce cytokines [13]. The mixture of **1** and **2** enhances the production of these cytokines in the wild-type mice but not in the *Cd1d*-deficient mice at a concentration of 5 µg/mL, demonstrating its selective activity. However, the amounts of cytokines produced were about 1/1000 of the mice treated at a concentration of 100 ng/mL of KRN7000. Therefore, the activity of the mixture of **1** and **2** was 1/50,000 of that of KRN7000. This result suggests that either calyxosides have very weak activity in enhancing the production of cytokines, or our preparation of calyxosides contains a very small amount of α-glycosides that are known to exhibit potent activity [12]. Further research is required to clarify the biological activity of calyxosides toward iNKT cells.

## 3. Discussion

Calyxoside B belongs to a group of two-headed glycosphingolipids which contain 2-amino-3-hydroxy functions in both ends, an *O*-glycoside at C-1 or C-3, and oxidation at C-18 [14]. The absolute configuration of each head group had exclusively been studied by analyzing the ECD data of the perbenzoate [15]. The position of the ketone was determined by mass spectrometry after Bayer-Villiger oxidation to a mixture of ester or reductive amination [5,6]. We have studied the absolute configuration of each head group of calyxoside B (**1**) and calyxoside (**2**) by the modified Mosher’s analysis in combination with conversion of the amino alcohol to an oxazolidinone. We came to a conclusion identical with those drawn in previous studies [14]. We applied the Beckmann rearrangement in determining the position of the ketone, which showed that C-18 was oxidized. Beckmann rearrangement of a ketoxime produces a mixture of isomeric amides, resulting in an introduction of a polar functional group in the middle of the long alkyl chain. Therefore, we anticipated that the biological activity of the products might be different from the starting material. In fact, the resulting mixture of amides showed a slightly diminished cytotoxic activity. Our preparation of calyxosides show weak activity in stimulating the NKT cells. Further study to elucidate the origin of this activity is in progress.

## 4. Materials and Methods

### 4.1. General Experimental Procedures

Optical rotation values were measured with a Jasco P-2200 polarimeter. NMR spectra were measured on a JEOL alpha 600 NMR spectrometer and referenced to solvent peaks: δ_H_ 3.30 and δ_c_ 49.0 for CD_3_OD. HRESI mass spectra were measured on a JEOL JMS-T100LC mass spectrometer or a Sciex Triple TOF5600 mass spectrometer. LC–MS experiments were performed on a Shimadzu LC-20AD solvent delivery pump with a COSMOSIL 2.5C_18_-MS-II (φ 2.0 × 100 mm) connected to a Bruker amaZon SL mass spectrometer.

Recycle HPLC was carried out on a Shimadzu LC-6AD pump with a Develosil C30-UG-5 column (φ 10 × 250 mm) and UV absorption was measured on a Shimadzu SPD-10A VP UV spectrometer. For other HPLC experiments, Shimadzu LC-20AT pumps with a COSMOSIL 5C_18_-MS-II column was used. The results of the MTT assay were recorded by Molecular Devices SPECTRA max M2.

### 4.2. Animal Material

The marine sponge *Cladocroce* sp. was collected by dredging at a depth of 245 m near the seamount Gonsone (29°45.599′ N, 129°23.754′ E), Japan, on 16 November 2008, during a cruse of R/V Nagasaki-maru. The sponge was frozen on site and kept frozen at −20 °C until extraction. The sponge is in a subglobular form with a smooth surface, and the color was dark brown in life. The ectosomal skeleton of the sponge could not be precisely observed because the surface of the sample used for identification was broken. The choanosomal skeleton consists of a multispicular bundle 60–620 µm in width, which forms a triangular, rectangular, hexagonal subcircular mesh with 1–2 mm in major axis. In between the bundles, unispicular isodictyal reticulations are present. Spicules are oxeas as megasclere with a slightly arched shaft at midpoint and sharply pointed extremities. The size of the oxea is 189.5–216.7 (200.9) µm in length and 8.4–10.4 (9.4) µm in width (*n* = 20). Microsclere is not present.

### 4.3. Extraction and Isolation

The sponge (100 g, wet weight) was homogenized and extracted with MeOH (200 mL × 2) and CHCl_3_-MeOH (1:1, 200 mL). The extracts were combined, concentrated, and partitioned between EtOAc and H_2_O. The EtOAc fraction was partitioned between *n*-hexane and 90% MeOH, and the H_2_O layer was extracted with *n*-BuOH. The 90% MeOH and *n*-BuOH fractions, which showed activity, were combined and subjected to ODS flash column chromatography with stepwise elution of 60%, 80%, 90%, 100% MeOH and CHCl_3_-MeOH (1:1). The first four fractions showed activity. They were combined and subjected to ODS HPLC with a gradient elution from 20% to 100% MeOH containing 0.5% acetic acid for 30 min to afford the mixture of **1** and **2**. To the mixture of **1** and **2** (43.6 mg) in H_2_O-acetone (1:1, 2 mL) were added Fmoc-OSu (45.2 mg) and Na_2_CO_3_ (7.1 mg). The solution was stirred for 24 h, and then acidified with HCl and the solvent was evaporated. The product was subjected to silica gel column chromatography with stepwise elution of 98:2, 9:1, 7:3 of CHCl_3_-MeOH, and CHCl_3_-MeOH-H_2_O (7:3:0.5). Further purification with 20 cycles of recycle HPLC using 90% MeOH as the mobile phase for 8 h permitted the separation of the Fmoc derivatives **3** and **4**. The Fmoc groups were remove by reaction with 20% piperidine in MeOH (2 mL) for 30 min. Purification of each product with HPLC (20%-100% MeCN in the presence of 0.5% AcOH) afforded calyxoside B (**1**, 5.3 mg) and calyxoside (**2**, 5.5 mg).

#### 4.3.1. Calyxoside B (**1**)

Colorless solid; ${\left[\alpha \right]}_{D}^{20}$ −11.3 (*c* 0.15, MeOH); ^1^H and ^13^C NMR (CD_3_OD), Table 1; HRESIMS calcd. for C_34_H_69_N_2_O_9_ [M + H]^+^ 649.5003, found 649.4982.

#### 4.3.2. Calyxoside (**2**)

Off-white solid; ${\left[\alpha \right]}_{D}^{20}$ −13.3 (*c* 0.15, MeOH); ^1^H NMR (CD_3_OD) 4.30 (d, *J* = 7.6 Hz, H1′), 4.0 (m, H1a), 3.92 (dd, *J* = 11.0 and 3.4 Hz, H1b), 3.89 (d, *J* = 11.0 Hz, H6′a), 3.77 (m, H3), 3.65 (dd, *J* = 11.7 and 6.2 Hz, H6′b), 3.43 (m, H26), 3.38 (m, H5′), 3.36 (m, H2), 3.32 (m, H3′), 3.27 (m, H4′), 3.23 (m, H2′), 3.07 (m, H27), 2.44 (m, H17), 2.44 (m, H19), 1.41 (m, H4), 1.38 (m, H25), 1.37–1.27 (m, H5 to H16, H20 to H24), 1.25 (m, H28). HRESIMS calcd. for C_34_H_69_N_2_O_9_ [M + H]^+^ 649.5003, found 649.4982.

### 4.4. Determination of the Absolute Configuration of the Sugar Moiety

The mixture of **1** and **2** (1.28 mg) was dissolved in 10% HCl in 50% MeOH and kept at 80 °C for 3 h. The solvent was evaporated and to the product were added L-cysteine methyl ester hydrochloride (12.5 mg) and pyridine (70 μL). The solution was kept at 60 °C for 1 h. Then phenyl isothiocyanate (12.5 μL) was added to the solution and the solution was kept at 60 °C for 1 h. The product was subjected to LC-MS using an ODS column (gradient elution of 10–15% MeCN for 60 min). The standards of D- and L-glucose, and D- and L-galactose, were derivatized in the same manner. Retention times of the standard samples (min): D-glucose (29.3), L-glucose (27.1), D-galactose (26.2), L-galactose (28.2). Retention times of the derivatives prepared from the mixture of **1** and **2** (26.2 and 29.3) (see Supplementary data).

### 4.5. Preparation of ***6*** and ***7***

The mixture of **3** and **4** (10.8 mg) was dissolved in 4 mL of MeOH. To the solution were added hydroxylamine (1.04 mg) and 50% KOH solution (3.5 µL). The solution was refluxed for 3 h and the solvent was evaporated. Cyanuric chloride (73.37 mg) and ZnCl_2_ (54.24 mg) in MeCN (1 mL) were added to the product. The solution was left standing at room temperature for 2 h. Purification of the reaction product with HPLC afforded the mixture of **6** and **7**. The mixture was subjected to methanolysis with 10% HCl in MeOH, and the product was dissolved in acetic anhydride (0.2 mL) containing pyridine (3 drops), and allowed to stand at room temperature for 1 h. The product was analyzed by LC-MS. Compound **6** was isolated after further purification of the mixture of **6** and **7** with HPLC. Compound **6** was analyzed in the same way. The LC-MS chromatograms are in Figure S14. The HRESIMS data of the mixture of **6** and **7**: calcd. for C_28_H_59_N_3_O_4_ [M + H]^+^ 502.4578, found 502.4579.

### 4.6. Modified Mosher’s Analysis

The mixture of **1** and **2** in 10% HCl in 50% MeOH was heated at 80 °C for 3 h. After removal of the solvent in vacuo, to the solution of the product in H_2_O-dioxane (300 μL) were added triethylamine (6.8 mL) and Boc-ON (8.64 mg). The solution was stirred at room temperature for 16 h. The solvent was evaporated, and the product was subjected to silica gel column chromatography by stepwise elution with CHCl_3_-MeOH (99:1 and 95:5) and CHCl_3_-MeOH-H_2_O (7:3:0.5). The CHCl_3_-MeOH (95:5) fraction was dried in vacuo. The product (5.0 mg) was dissolved in pyridine (150 µL) to which was added pivaloyl chloride (1.5 µL) and kept at rt with the reaction monitored by TLC. The reaction was completed after the addition of 4.5 µL of pivaloyl chloride in total. The solvent was evaporated and partitioned between H_2_O and ethyl acetate. The EtOAc layer (1.8 mg) that contained **12** was treated with (*R*)-MTPA chloride (10 µL) in pyridine (25 µL) at room temperature for 16 h. The product was dried and partitioned between H_2_O and CHCl_3_. The organic layer was dried to yield the (*S*)-MTPA ester (**13**). The (*R*)-MTPA ester (**14**) was prepared in the same way.

**13**: ^1^H NMR (CD_3_OD) 5.19 (m, H-3), 5.06 (m, H-26), 4.05 (H-2), 4.01 (H-1b), 3.82 (H-1a, H-27), 1.70 (H-4), 1.52 (H_2_-25), 1.10 (H_3_-28); HRESIMS calcd. for C_63_H_96_N_2_O_13_F_6_ [M + Na]^+^ 1225.6709, found 1225.6721.

**14**: ^1^H NMR (CD_3_OD) 5.14 (m, H-3), 5.09 (m, H-26), 4.13 (H-2, H-1b), 3.96 (H-1a), 3.81 (H-27), 1.60 (H_2_-4), 1.62 (H_2_-25), 0.96 (H_3_-28); HRESIMS calcd. for C_63_H_96_N_2_O_13_F_6_ [M + Na]^+^ 1225.6709, found 1225.6733.

### 4.7. Preparation of ***15***

Compound **12** (1.8 mg) was dissolved in a mixture of TFA (0.1 mL) and CH_2_Cl_2_ (0.5 mL) and left standing at room temperature for 1 h. After evaporation of the solvent, the mixture was dissolved in 1 M NaOH (2 mL) and extracted with CH_2_Cl_2_ (2 mL x 2). The combined organic layer was evaporated. To the residue dissolved in in DMF (150 µL) was added *N*, *N*’-carbonyldiimidazole (1.0 mg) and it was kept at 90 °C for 1 h. The reaction mixture was subjected to HPLC to obtain **15**.

**15**: ^1^H-NMR (CD_3_OD) 4.68 (m, H-3), 4.23 (dd, J = 12.1, 3.6, H1a), 4.10 (m, H-26), 4.04 (m, H-1b), 3.97 (m, H-2), 3.56 (m, H-27), 2.44 (m, H_2_-17, H_2_-19); HRESIMS calcd. for C_35_H_62_N_2_O_7_ [M + Na]^+^ 645.4449, found 645.4454.

### 4.8. Cytotoxicity Assay

HeLa human cervical cancer cells were purchased from the RIKEN BRC through the National Bio-Resource Project of the MEXT/AMED, Japan. The cells were cultured in DMEM, containing 10% fetal bovine serum and 1% Penicillin-Streptomycin Solution (×100) (FUJIFILM-Wako Pure Chemical Co., Osaka, Japan), at 37 °C under an atmosphere of 5% CO_2_. To each well of a 96-well microplate containing cell suspension (1 × 10^4^ cells/mL, 200 µL) was added a sample dissolved in MeOH after a preincubation for 24 h, and the plate was incubated for 48 h. Then, 3-(4,5-dimethylthiazol-2-yl)-2,5-diphenyltetrazolium bromide (MTT) in PBS (1 mg/mL, 50 μL) was added to each well, and the plate was further incubated for 3 h. After removing the supernatant, the residue was dissolved in DMSO (150 μL). The absorbance at 538 nm was measured. Three replicates were examined to determine the IC_50_.

### 4.9. Analysis of Cytokine Production from Mouse Spleen Cells

C57BL/6 wild type mice were purchased from Clea Japan, Inc. *Cd1d*-deficientmice [16] were kindly provided by Dr. Luc van Kaer (Nashville, TN, USA). Mice were kept under specific pathogen-free conditions and were used at 8–16 wk of age. All experiments were in accordance with protocols approved by the Animal Care and Use Committee of Kanazawa University.

Spleen cells were collected and used after treatment with red blood cell lysing buffer (Merck KGaA). RPMI 1640 (Merck KGaA) containing 10% heat-inactivated fetal calf serum (Invitrogen), 5 × 10^−5^ M 2-mercaptoethanol, 2mM L-glutamine, 25 mM 4-(2-hydroxyethyl)-1-piperazine ethanesulphonic acid (Mediatech, Manassas, VA, USA), 100 U/mL penicillin, and 100 μg/mL streptomycin was used as the culture medium. Spleen cells with single cell suspension were cultured with or without the 3:1 mixture of calyxoside B and calyxoside in the concentration of 10^5^/100 μL. For the measurement of cytokine production, the culture supernatants were collected 72 h post incubation. Cytokines in culture supernatants were analyzed by cytometric bead array (BD Biosciences) according to the manufacturer’s protocol.

## Figures and Tables

**Table 1:** ^1^H NMR (600 MHz) and ^13^C NMR (150 MHz) data for calyxoside B (**1**) in CD_3_OD.

No.	δ_C_, Type	δ_H_, Mult (*J* in Hz)	COSY	HMBC (Carbon Number)
1	67.0, CH_2_	3.97 m	H-2	2, 1′
2	57.0, CH	3.33 m	H-1, H-3	
3	70.3, CH	3.76 m	H-2, H2-4	
4	34.2, CH_2_	1.46 m, 1.51 m		5
5	27.1, CH_2_	1.34 m, 1.53 m		
6–15	30.2–30.8, CH_2_	1.25–1.35 m		
16	24.9, CH_2_	1.54 m	H-17	17, 18
17	43.5, CH_2_	2.44 m	H-16	15, 16, 18
18	214.4, C			
19	43.5, CH_2_	2.44 m	H-20	18, 20, 21
20	24.9, CH_2_	1.54 m	H-19	18, 19
21–23	30.2–30.8, CH_2_	1.25–1.35 m		
24	26.3, CH_2_	1.38 m, 1.51 m		25
25	34.6, CH_2_	1.39 m, 1.54 m		24
26	73.2, CH	3.42 m	H2-25, H-27	
27	53.4, CH	3.08 m	H-26, H3-28	26, 28
28	16.0, CH_3_	1.25 d (6.7)	H-27	26, 27
1′	104.6, CH	4.28 d (7.6)	H-2′	1
2′	72.4, CH	3.52 dd (7.7)	H-1′, H-3′	1′, 3′
3′	74.8, CH	3.48 dd (9.7, 3.3)	H-2′, H-4′	2′
4′	70.4, CH	3.81 d (3.3)	H-3′	2′, 3′
5′	77.0, CH	3.55 m	H2-6′	6′
6′a	62.7, CH_2_	3.72 dd (11.4, 4.5)	H-5′, H-6′b	
6′b		3.76 m	H-5″, H-6′a	5′

## Data Availability

All data is contained within this article and Supplementary Materials.

## Supplementary Materials

The following are available online at https://www.mdpi.com/article/10.3390/md19060287/s1, Figure S1: Photograph of a marine sponge *Cladocroce* sp., Figure S2: ^1^H NMR spectrum (600 MHz) of calyxoside B (**1**) in CD_3_OD, Figure S3: COSY spectrum (600 MHz) of calyxoside B (**1**) in CD_3_OD, Figure S4: ^13^C NMR spectrum (150 MHz) of calyxoside B (**1**) in CD_3_OD, Figure S5: HSQC spectrum (600 MHz) of calyxoside B (**1**) in CD_3_OD, Figure S6: HMBC spectrum (600 MHz) of calyxoside B (**1**) in CD_3_OD, Figure S7: ^1^H NMR spectrum (600 MHz) of calyxoside (**2**) in CD_3_OD, Figure S8: COSY spectrum (600 MHz) of calyxoside (**2**) in CD_3_OD, Figure S9: ^13^C NMR spectrum (150 MHz) of calyxoside (**2**) in CD_3_OD, Figure S10: HSQC spectrum (600 MHz) of calyxoside (**2**) in CD_3_OD, Figure S11: HMBC spectrum (600 MHz) of calyxoside (**2**) in CD_3_OD, Figure S12: ^1^H NMR spectrum (600 MHz) of the aglycone (**5**) in CD_3_OD, Figure S13: COSY spectrum (600 MHz) of the aglycone (**5**) in CD_3_OD, Figure S14: LC–MS chromatograms of derivatives of amide methanolysis products, Figure S15: ^1^H NMR spectrum (600 MHz) of the (S)-MTPA ester (**13**) in CD_3_OD, Figure S16: COSY spectrum (600 MHz) of the (S)-MTPA ester (**13**) in CD_3_OD, Figure S17: HSQC spectrum (600 MHz) of the (S)-MTPA ester (**13**) in CD_3_OD, Figure S18: ^1^H NMR spectrum (600 MHz) of the (R)-MTPA ester (**14**) in CD_3_OD, Figure S19: COSY spectrum (600 MHz) of the (R)-MTPA ester (**14**) in CD_3_OD, Figure S20: HSQC spectrum (600 MHz) of the (R)-MTPA ester (**14**) in CD_3_OD, Figure S21: ^1^H NMR spectrum (600 MHz) of the bis-oxazolidinone (**15**) in CD_3_OD, Figure S22: COSY spectrum (600 MHz) of the bis-oxazolidinone (**15**) in CD_3_OD, Figure S23: NOESY spectrum (600 MHz) of the bis-oxazolidinone (**15**) in CD_3_OD, Figure S24: LC–MS chromatograms of sugar derivatives, Figure S25: Graphs of MTT assay of **1**, **2**, **5** and the mixture of **6** and **7** against HeLa cells.
