# Supplementary material for: Structure Elucidation of Calyxoside B, a Bipolar Sphingolipid from a Marine Sponge Cladocroce sp. through the Use of Beckmann Rearrangement
Source: Mar Drugs. 2021 May 21;19(6):287. doi: 10.3390/md19060287 (PMC8224005; doi:10.3390/md19060287)
Supplement: Supplementary file 1 [file marinedrugs-19-00287-s001.zip › marinedrugs-1232621-supplementary.pdf]

Supplementary Material

Structure elucidation of calyxoside B, a bipolar sphingolipid from a marine sponge *Cladocroce* sp. through the use of Beckmann rearrangement

Kenji Sugawara [1], Hiroshi Watarai [2], Yuji Ise [3], Hisayoshi Yokose [4], Yasuhiro Morii [5], Nobuhiro Yamawaki [6], ^Shigeru Okada [7], Shigeki Matsunaga [8,*]

[1] Laboratory of Aquatic Natural Products Chemistry, Graduate School of Agricultural and Life Sciences, The University of Tokyo, 1-1-1 Yayoi, Bunkyo-ku, Tokyo 113-8657, Japan

[2] Department of Immunology and Stem Cell Biology, Faculty of Medicine, Kanazawa University, Ishikawa 920-8640, Japan

[3] Sesoko Station, Tropical Biosphere Research Center, University of the Ryukyus, 3422 Sesoko, Motobu, Okinawa 905-0227, Japan

[4] Graduate School of Science and Technology, Kumamoto University, 2-39-1 Kurokami, Chuo-ku, Kumamoto 860-8555, Japan

[5] Graduate School of Fisheries Science and Environmental Studies, Nagasaki University, Nagasaki 852-8521, Japan

[6] Graduate School of Fisheries Science and Environmental Studies, Nagasaki University, Nagasaki 852-8521, Japan

[7] Laboratory of Aquatic Natural Products Chemistry, Graduate School of Agricultural and Life Sciences, The University of Tokyo, 1-1-1 Yayoi, Bunkyo-ku, Tokyo 113-8657, Japan

[8] Laboratory of Aquatic Natural Products Chemistry, Graduate School of Agricultural and Life Sciences, The University of Tokyo, 1-1-1 Yayoi, Bunkyo-ku, Tokyo 113-8657, Japan

Table of contents

Figure S1. Photograph of a marine sponge *Cladocroce* sp.
Figure S2. ^{1}H NMR spectrum (600 MHz) of calyxoside B (**1**) in CD_3OD,
Figure S3. COSY spectrum (600 MHz) of calyxoside B (**1**) in CD_3OD
Figure S4. ^{13}C NMR spectrum (150 MHz) of calyxoside B (**1**) in CD_3OD
Figure S5. HSQC spectrum (600 MHz) of calyxoside B (**1**) in CD_3OD
Figure S6. HMBC spectrum (600 MHz) of calyxoside B (**1**) in CD_3OD
Figure S7. ^{1}H NMR spectrum (600 MHz) of calyxoside (**2**) in CD_3OD
Figure S8. COSY spectrum (600 MHz) of calyxoside (**2**) in CD_3OD
Figure S9. ^{13}C NMR spectrum (150 MHz) of calyxoside (**2**) in CD_3OD
Figure S10. HSQC spectrum (600 MHz) of calyxoside (**2**) in CD_3OD
Figure S11. HMBC spectrum (600 MHz) of calyxoside (**2**) in CD_3OD
Figure S12. ^{1}H NMR spectrum (600 MHz) of the aglycone (**5**) in CD_3OD
Figure S13. COSY spectrum (600 MHz) of the aglycone (**5**) in CD_3OD
Figure S14. LC–MS chromatograms of derivatives of amide methanolysis products
Figure S15. ^{1}H NMR spectrum (600 MHz) of the (S)-MTPA ester (**13**) in CD_3OD
Figure S16. COSY spectrum (600 MHz) of the (S)-MTPA ester (**13**) in CD_3OD
Figure S17. HSQC spectrum (600 MHz) of the (S)-MTPA ester (**13**) in CD_3OD
Figure S18. ^{1}H NMR spectrum (600 MHz) of the (R)-MTPA ester (**14**) in CD_3OD
Figure S19. COSY spectrum (600 MHz) of the (R)-MTPA ester (**14**) in CD_3OD
Figure S20. HSQC spectrum (600 MHz) of the (R)-MTPA ester (**14**) in CD_3OD
Figure S21. ^{1}H NMR spectrum (600 MHz) of the bis-oxazolidinone (**15**) in CD_3OD
Figure S22. COSY spectrum (600 MHz) of the bis-oxazolidinone (**15**) in CD_3OD
Figure S23. NOESY spectrum (600 MHz) of the bis-oxazolidinone (**15**) in CD_3OD
Figure S24. LC–MS chromatograms of sugar derivatives
Figure S25. Graphs of MTT assay of **1**, **2**, **5** and the mixture of **6** and **7** against HeLa cells

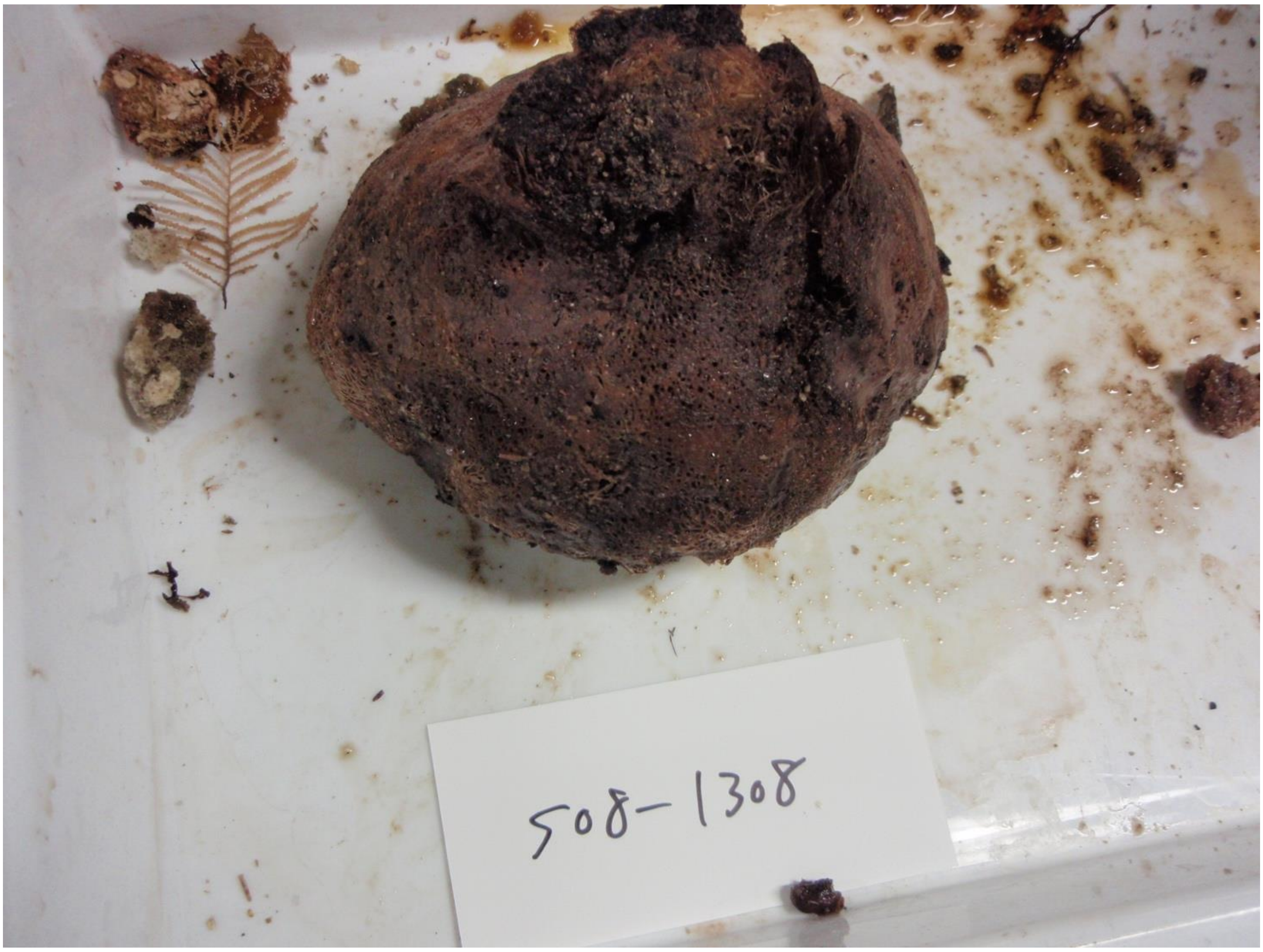

Figure S1. Photograph of a marine sponge *Cladocroce* sp.

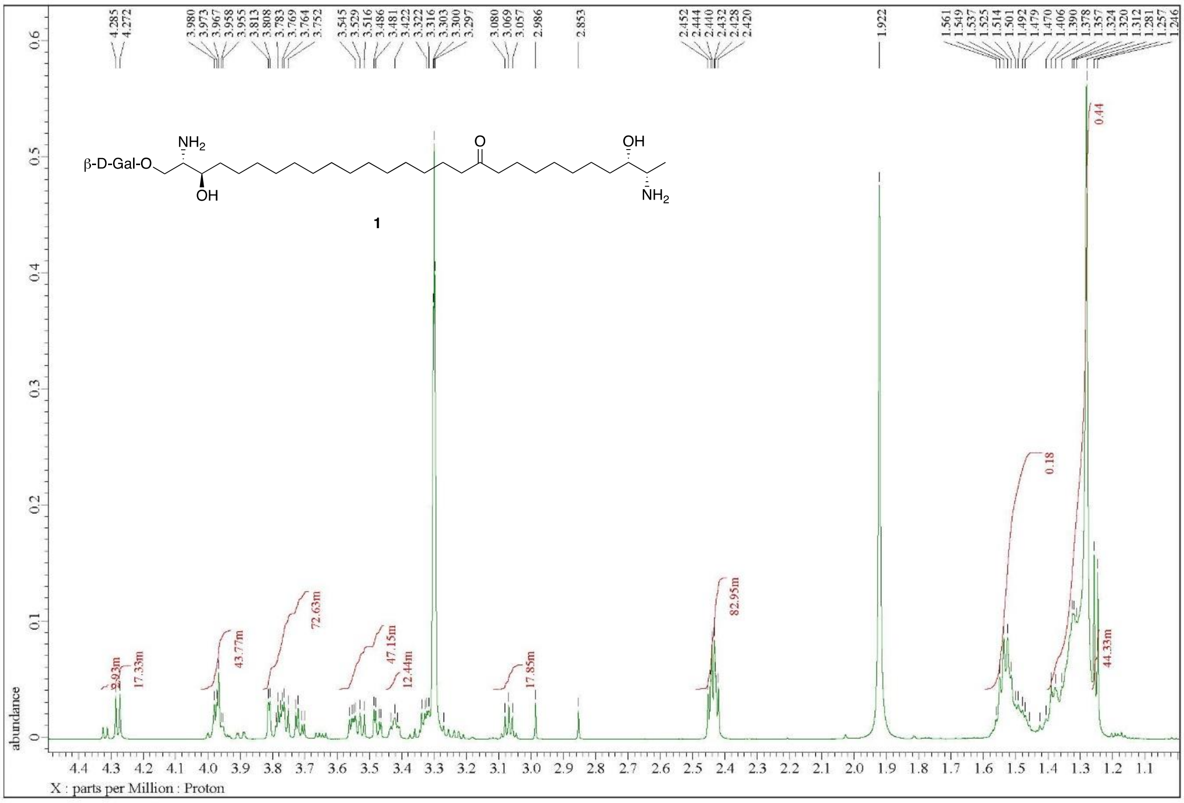

Figure S2. ^{1}H NMR spectrum (600 MHz) of calyxoside B (**1**) in CD_3OD

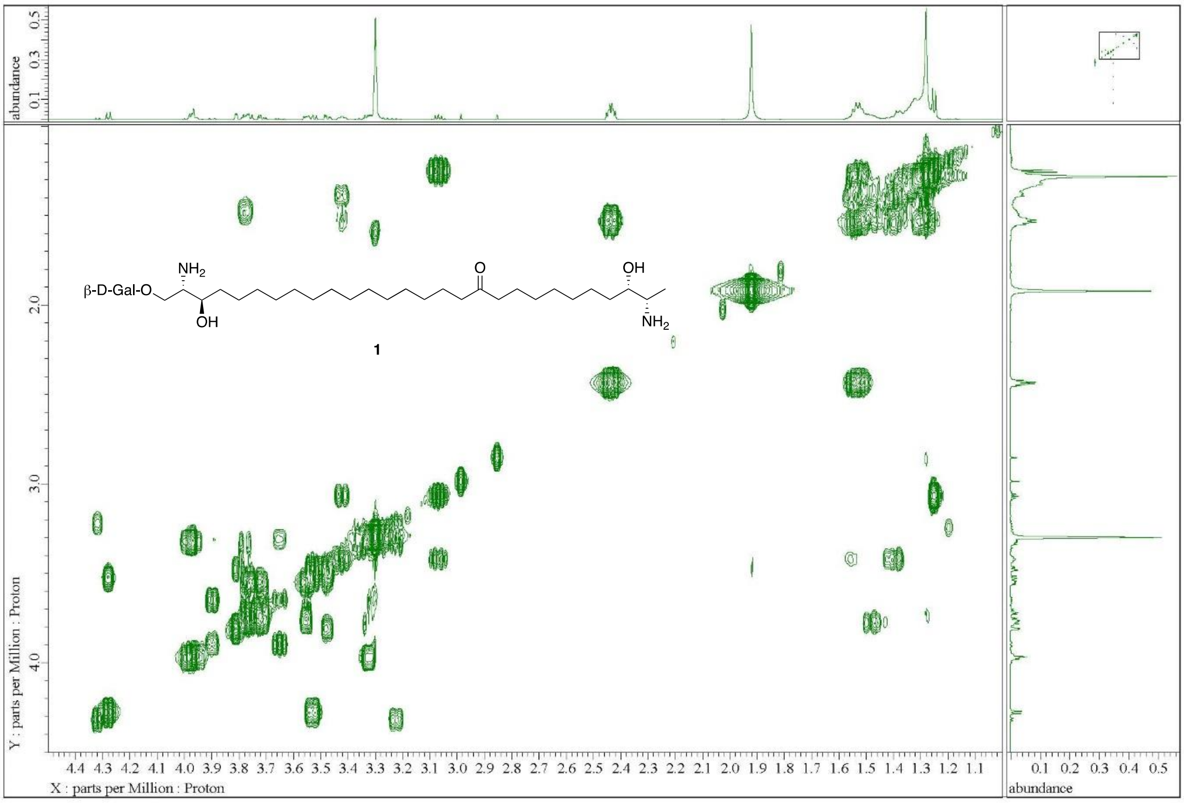

Figure S3. COSY spectrum (600 MHz) of calyxoside B (**1**) in CD_3OD

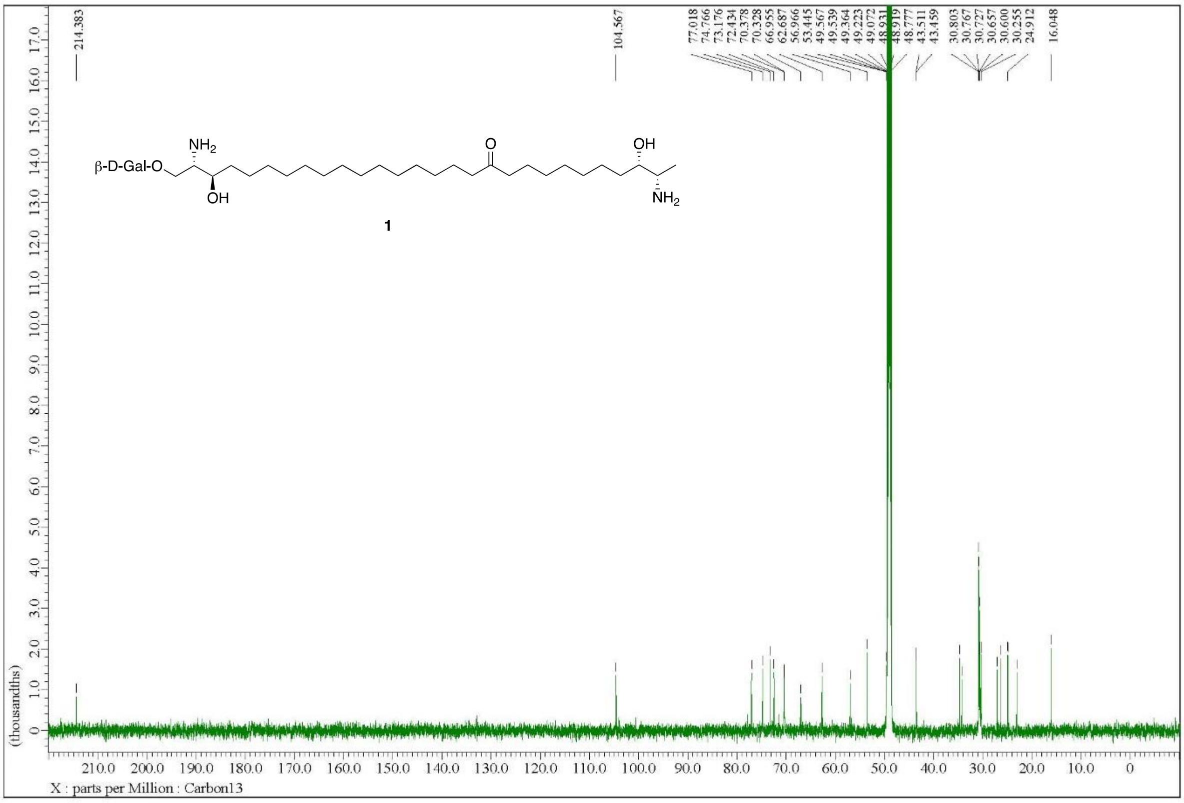

Figure S4. ^{13}C NMR spectrum (150 MHz) of calyxoside B (**1**) in CD_3OD

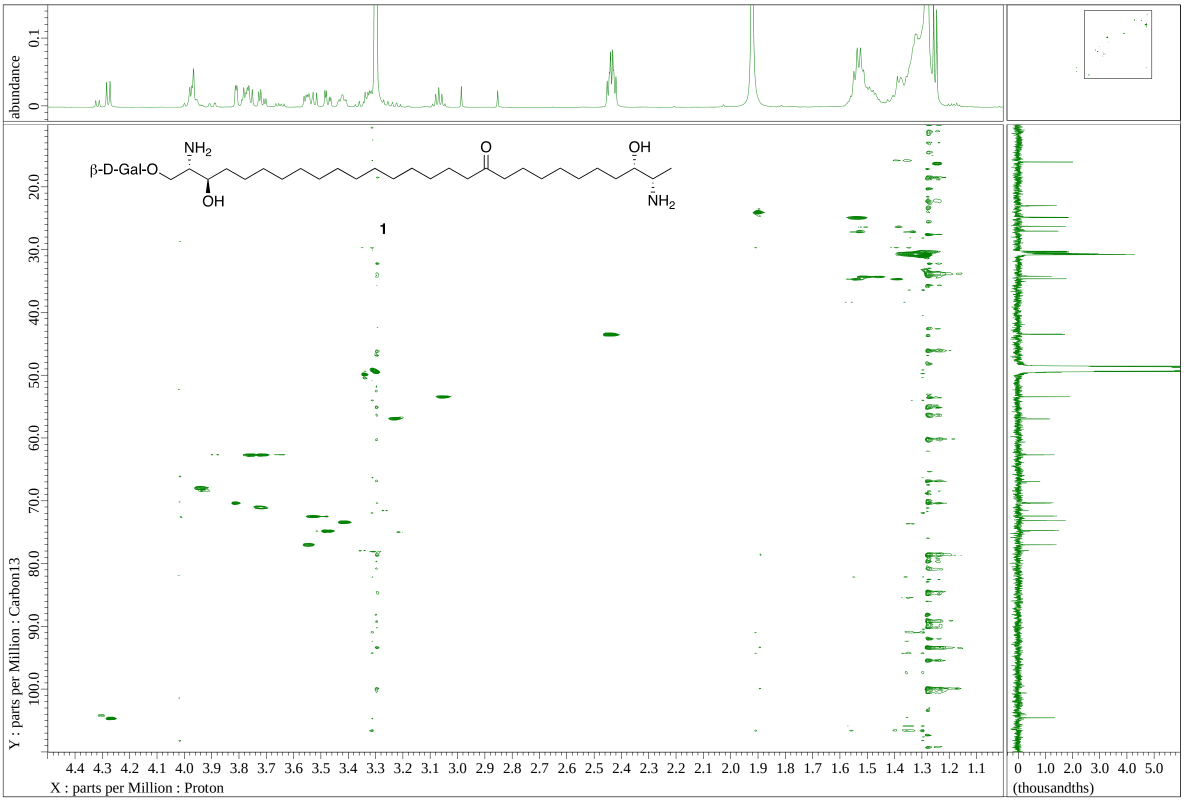

Figure S5. HSQC spectrum (600 MHz) of calyxoside B (**1**) in CD_3OD

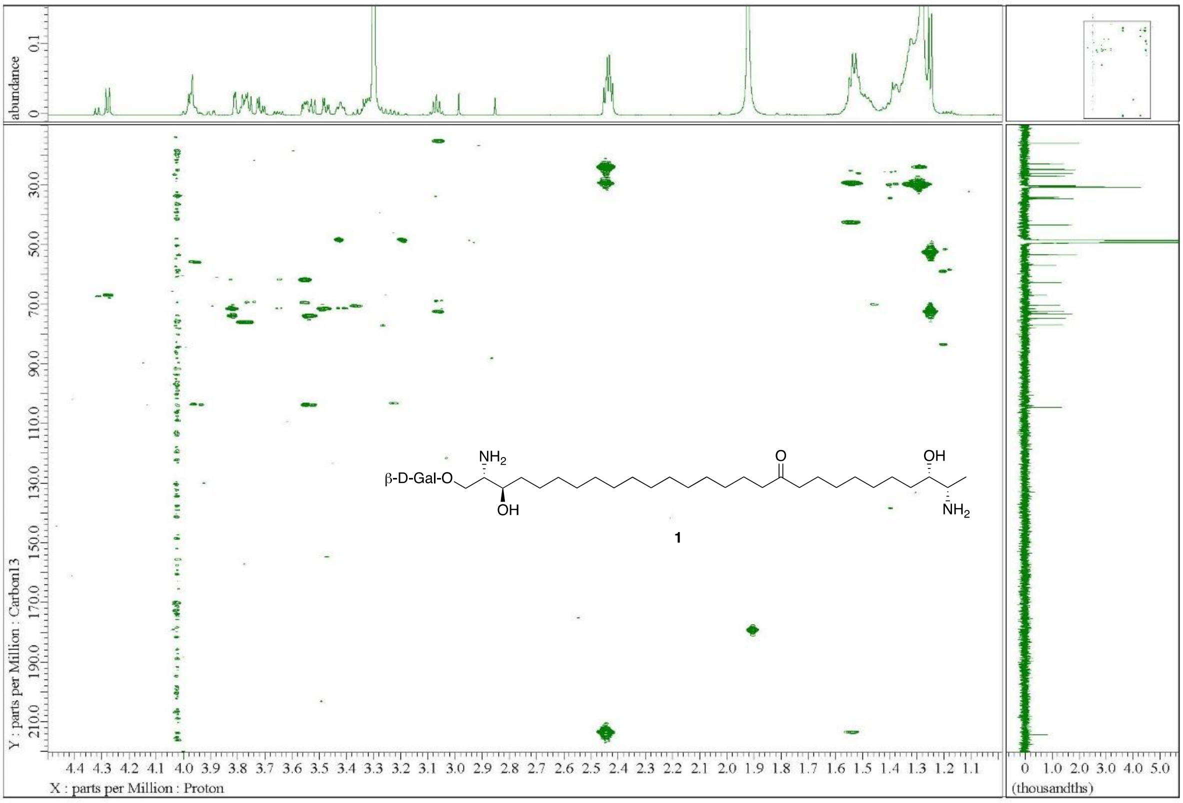

Figure S6. HMBC spectrum (600 MHz) of calyxoside B (**1**) in CD_3OD

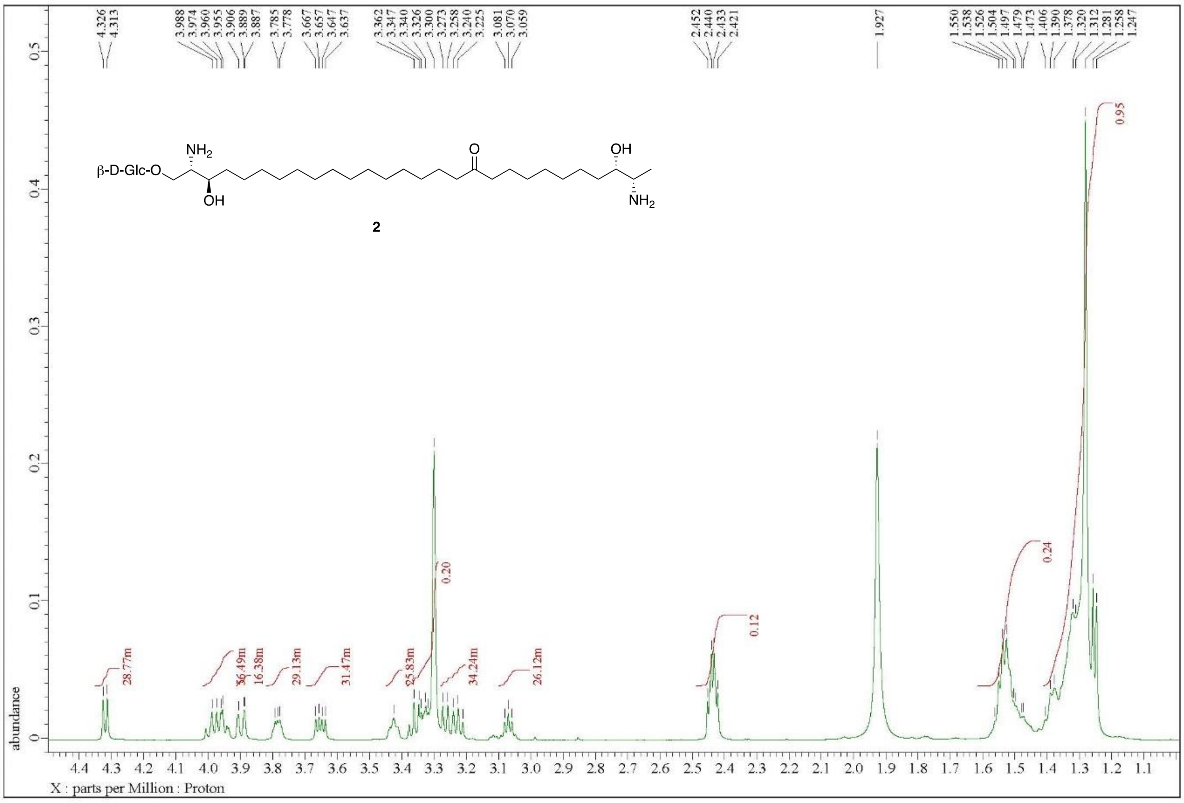

Figure S7. ^{1}H NMR spectrum (600 MHz) of calyxoside (**2**) in CD_3OD

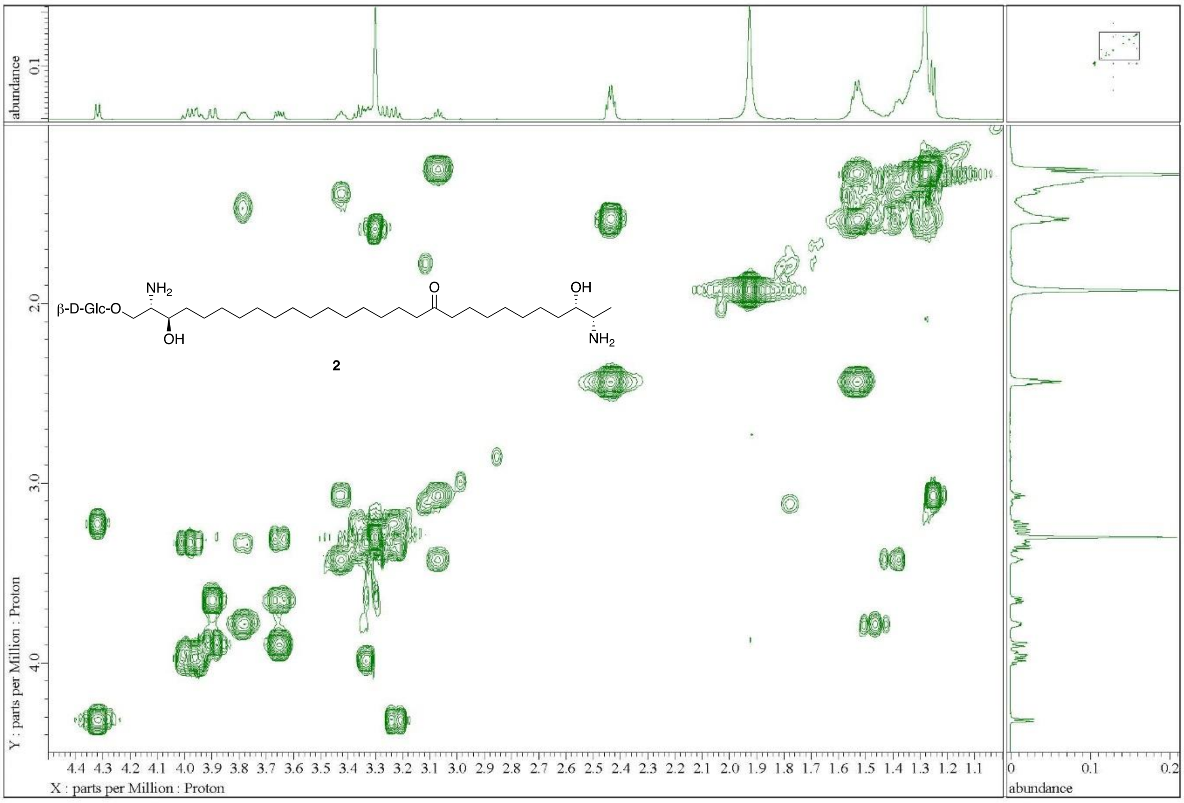

Figure S8. COSY spectrum (600 MHz) of calyxoside (**2**) in CD_3OD

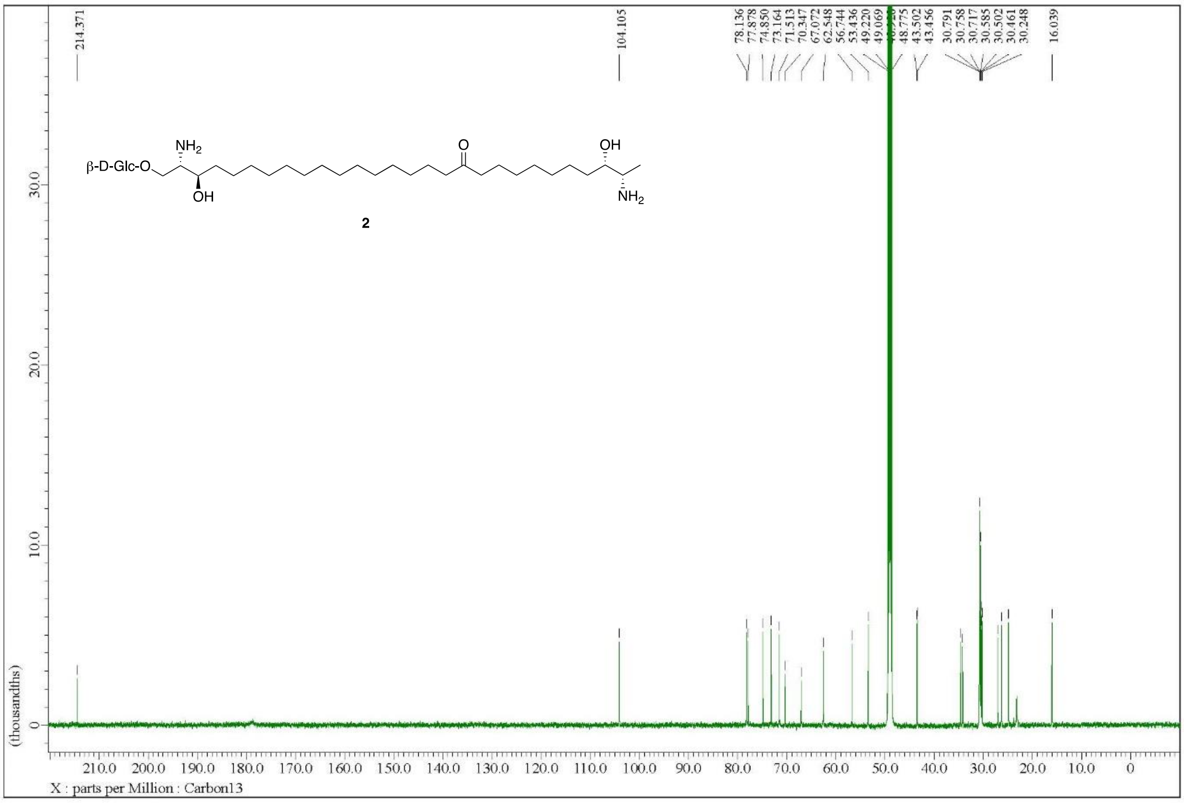

Figure S9. ^{13}C NMR spectrum (150 MHz) of calyxoside (**2**) in CD_3OD

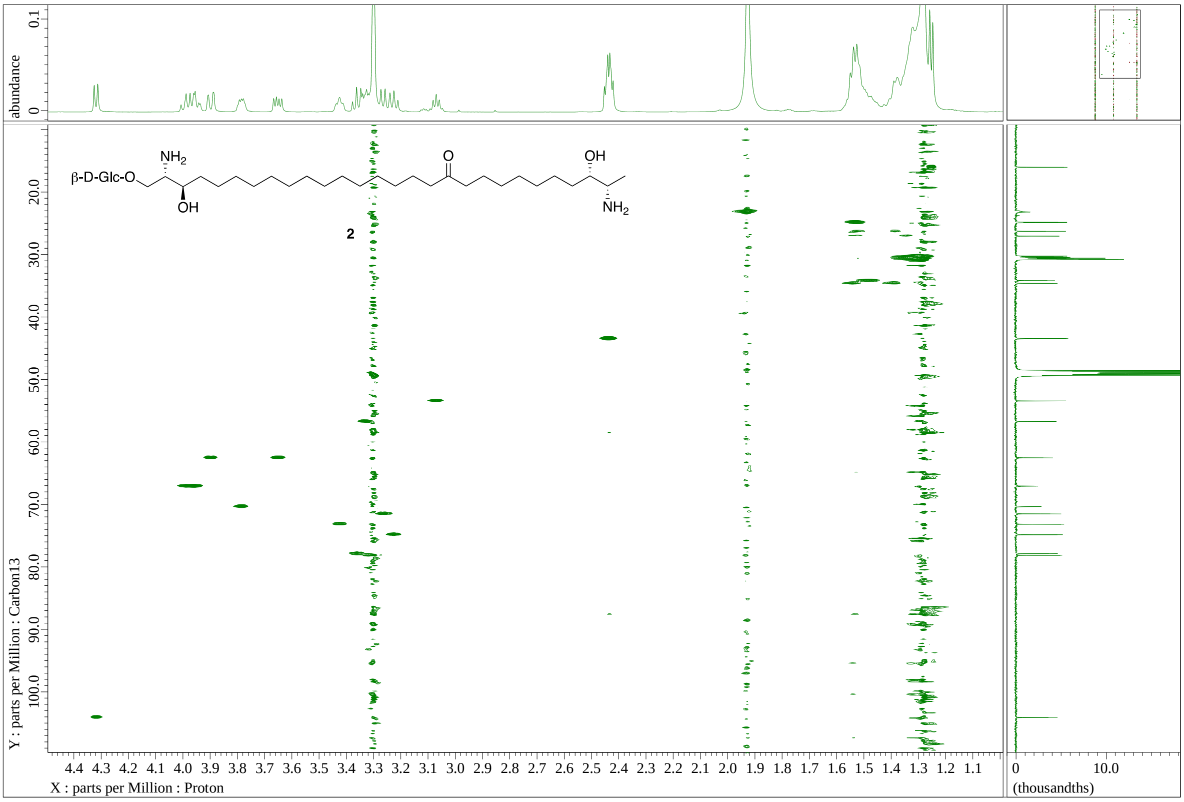

Figure S10. HSQC spectrum (600 MHz) of calyxoside (**2**) in CD_3OD

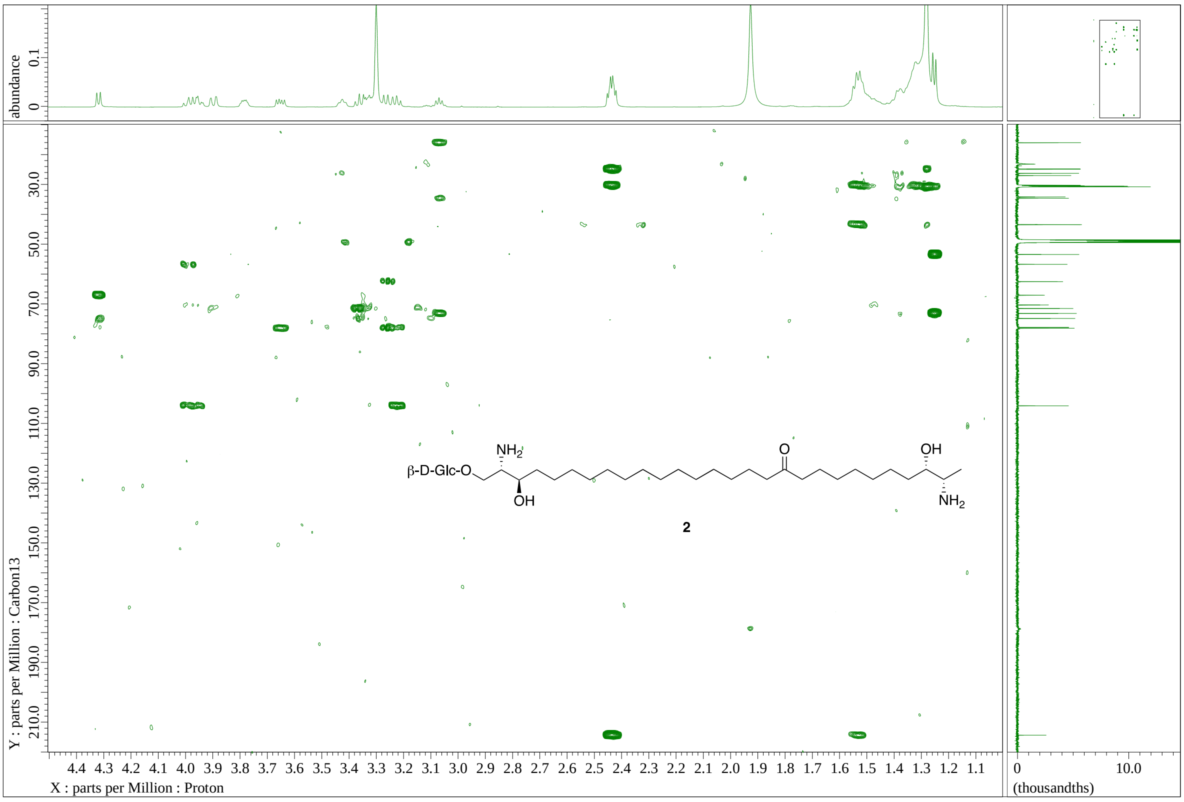

Figure S11. HMBC spectrum (600 MHz) of calyxoside (**2**) in CD_3OD

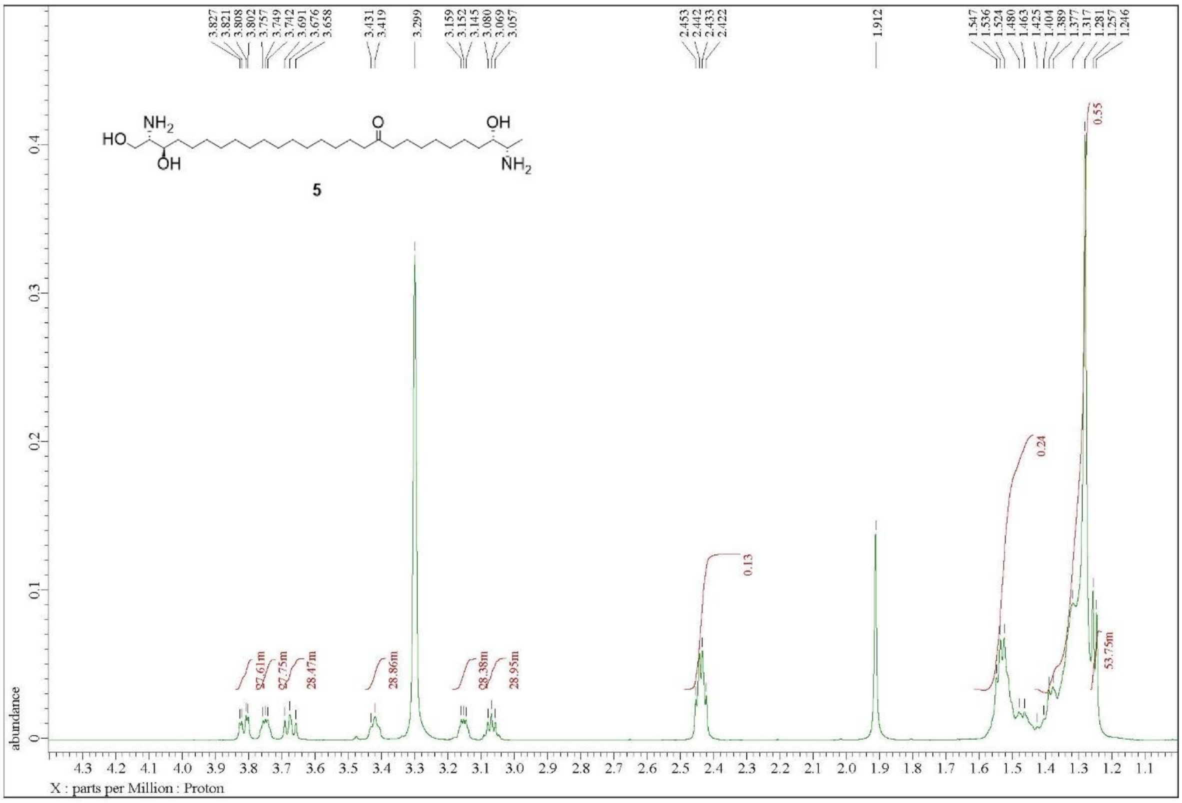

Figure S12. ^{1}H NMR spectrum (600 MHz) of the aglycone (**5**) in CD_3OD

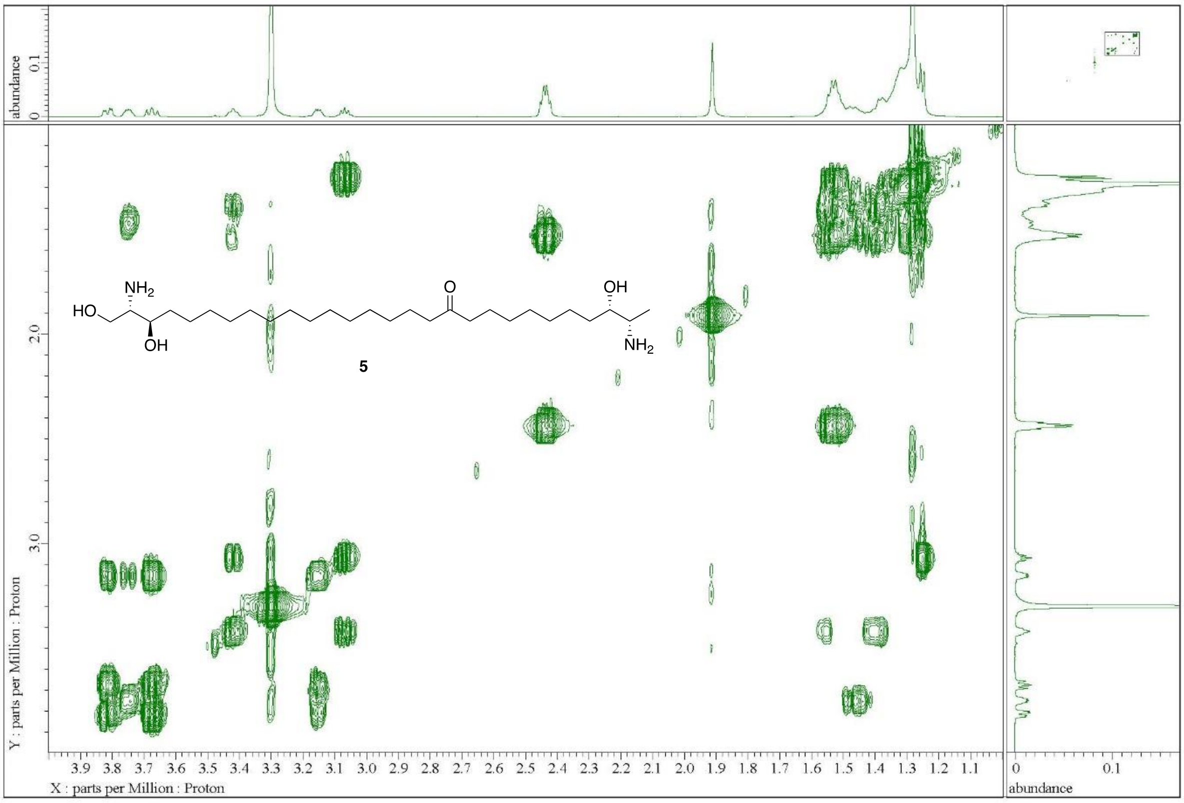

Figure S13. COSY spectrum (600 MHz) of the aglycone (**5**) in CD_3OD

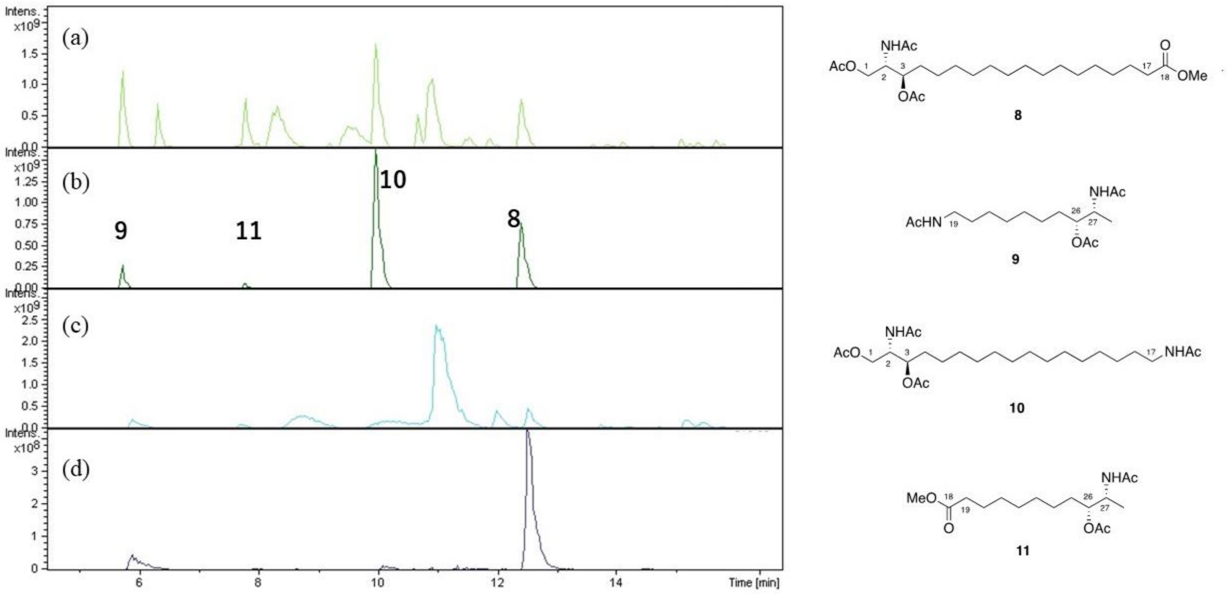

Figure S14. LC–MS chromatograms of derivatives of amide methanolysis products
a: All ions from **6** and **7**, b: Ions at *m/z* 472.3 (**8**), 315.2 (**9**), 471.3 (**10**) and 316.2 (**11**) from **6** and **7**, c: All ions from **6**, d: Ions at *m/z* 472.3, 315.2, 471.3 and 316.2 from **6**

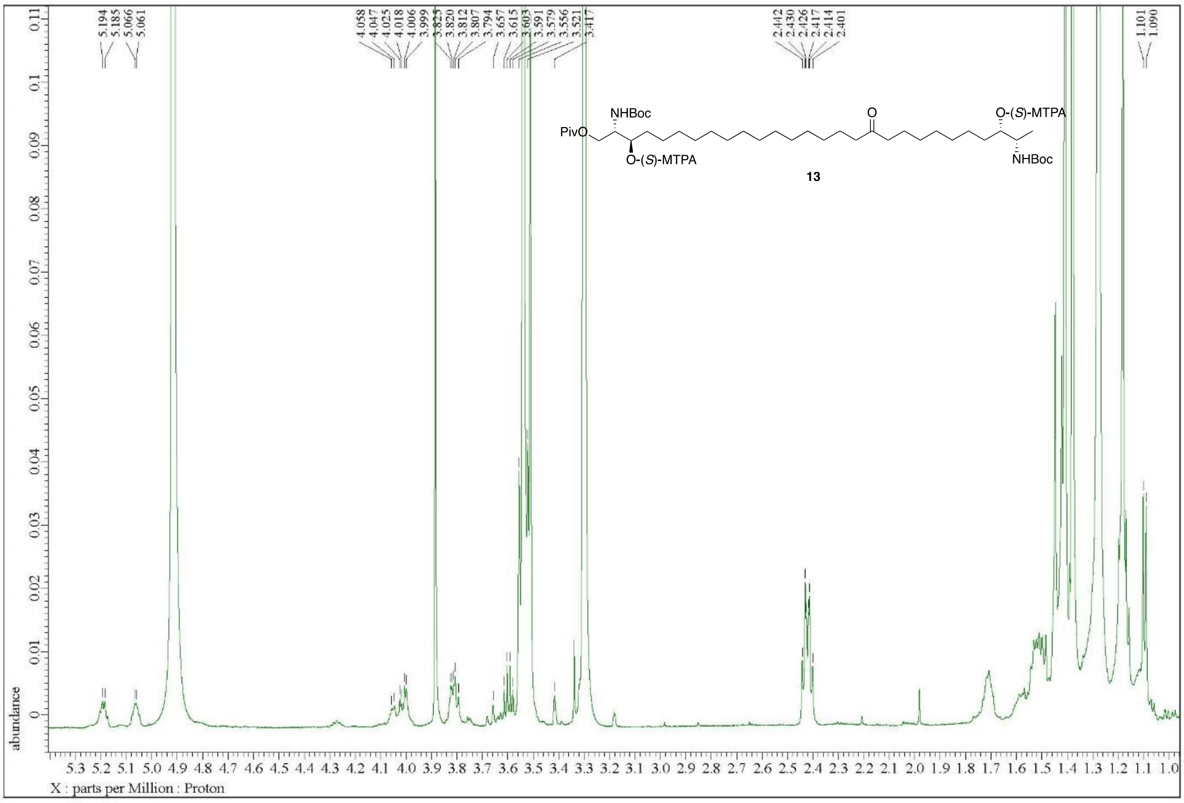

Figure S15. ^{1}H NMR spectrum (600 MHz) of the (*S*)-MTPA ester (**13**) in CD_3OD

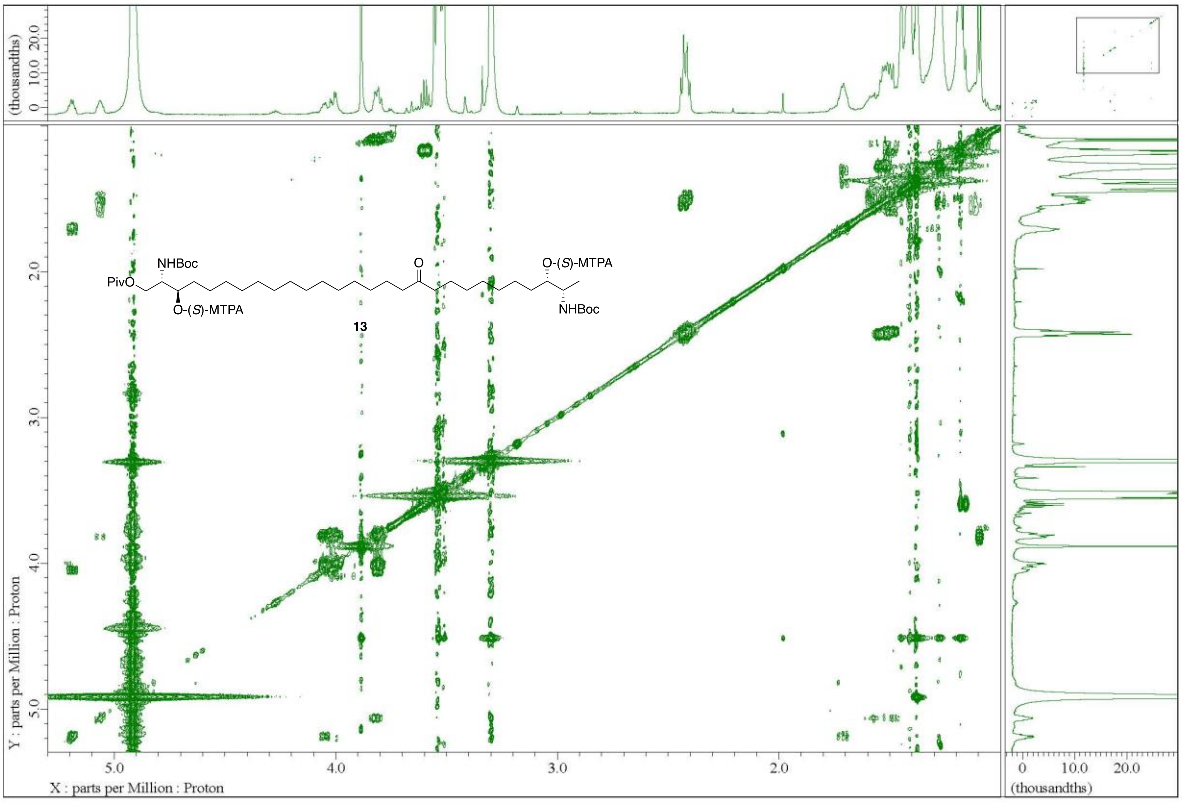

Figure S16. COSY spectrum (600 MHz) of the (*S*)-MTPA ester (**13**) in CD_3OD

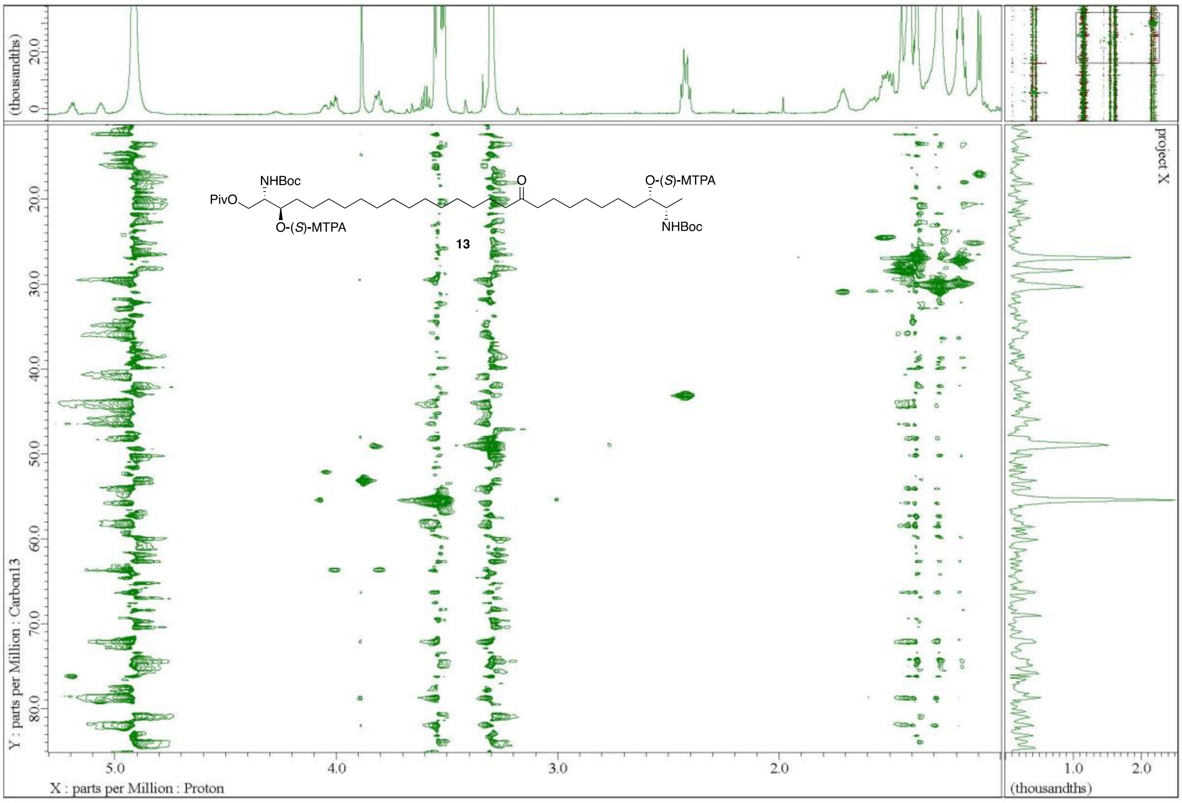

Figure S17. HSQC spectrum (600 MHz) of the (*S*)-MTPA ester (**13**) in CD_3OD

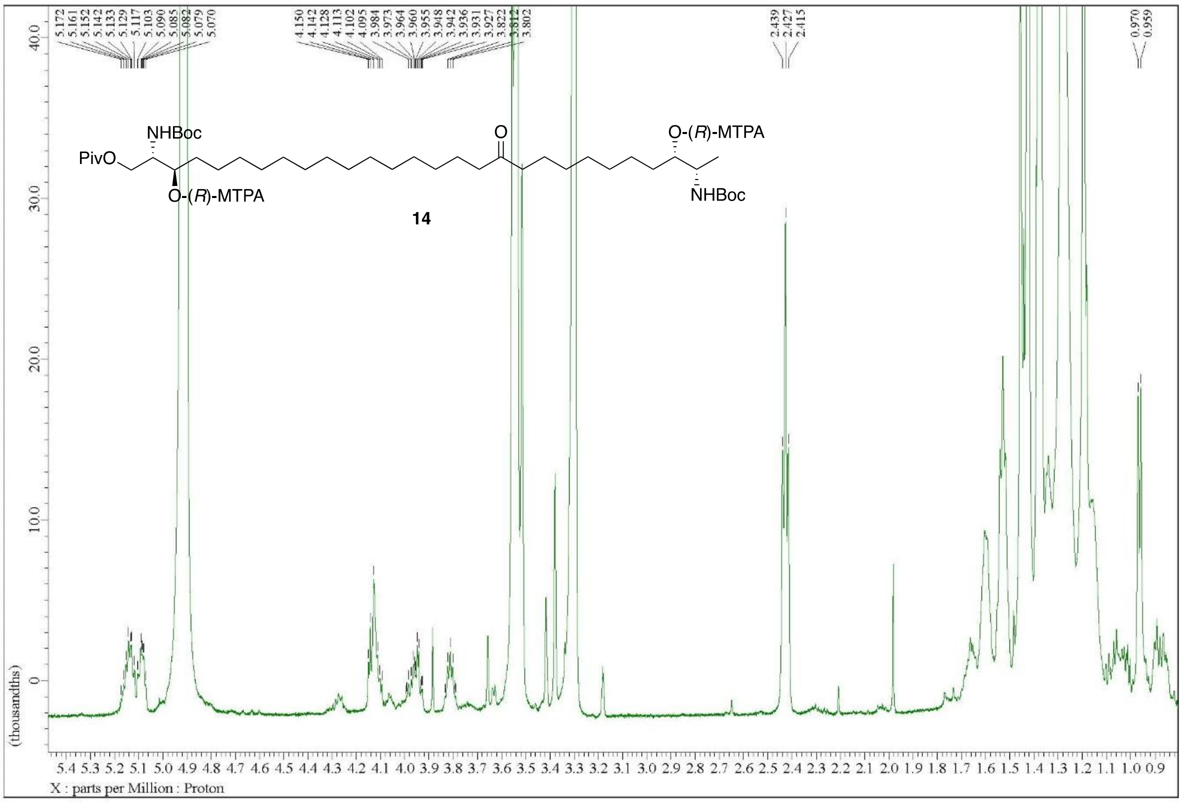

Figure S18. ^{1}H NMR spectrum (600 MHz) of the (*R*)-MTPA ester (**14**) in CD_3OD

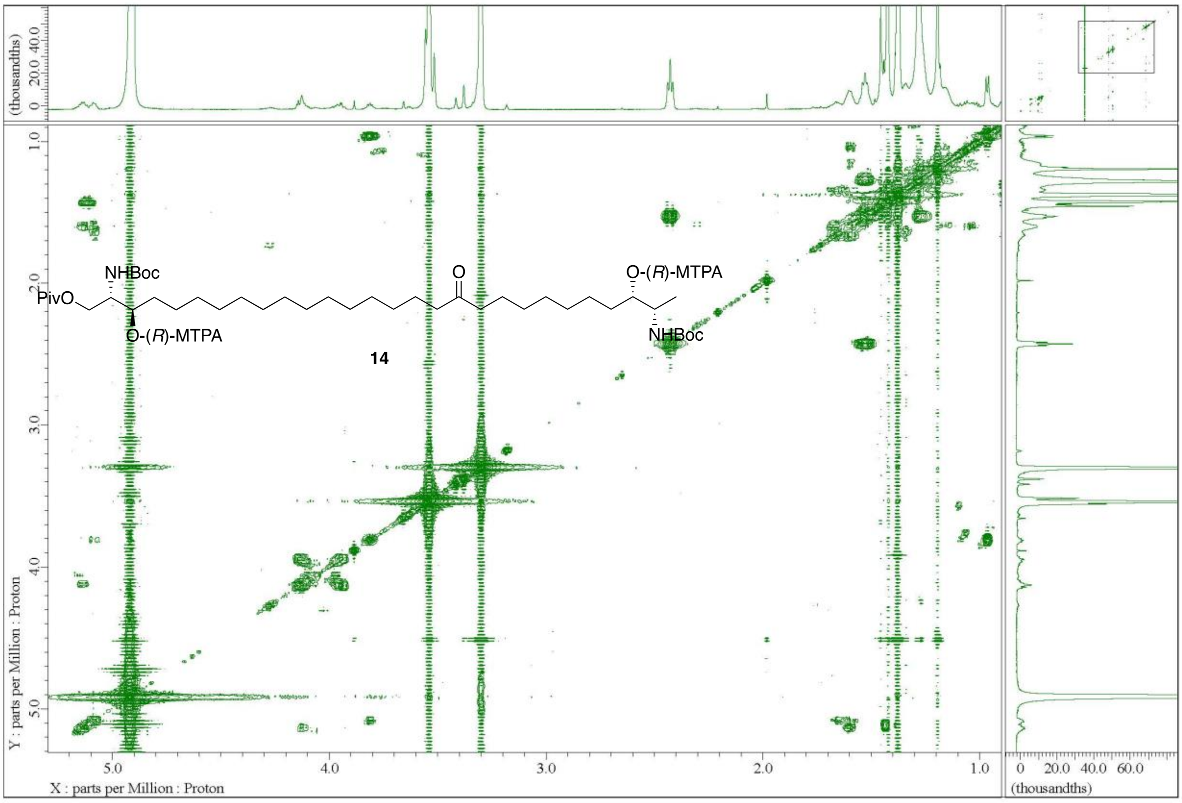

Figure S19. COSY spectrum (600 MHz) of the (*R*)-MTPA ester (**14**) in CD_3OD

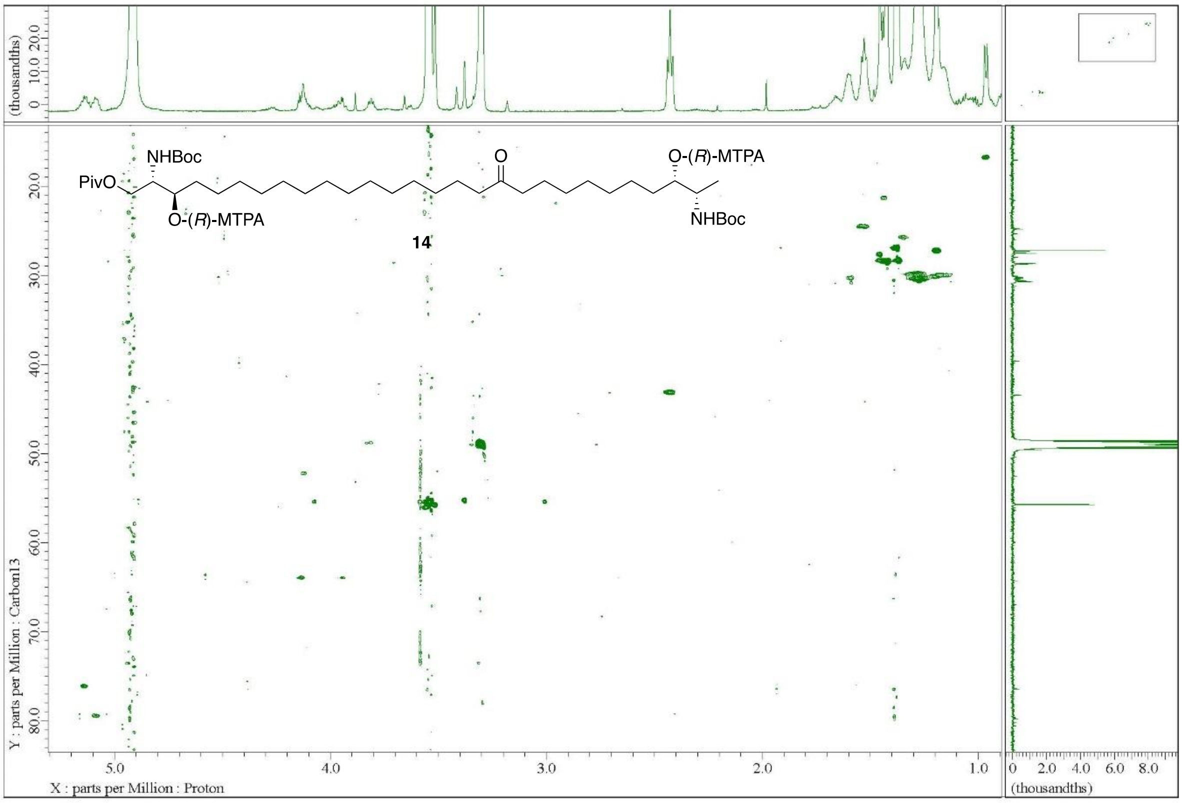

Figure S20. HSQC spectrum (600 MHz) of the (*R*)-MTPA ester (**14**) in CD_3OD

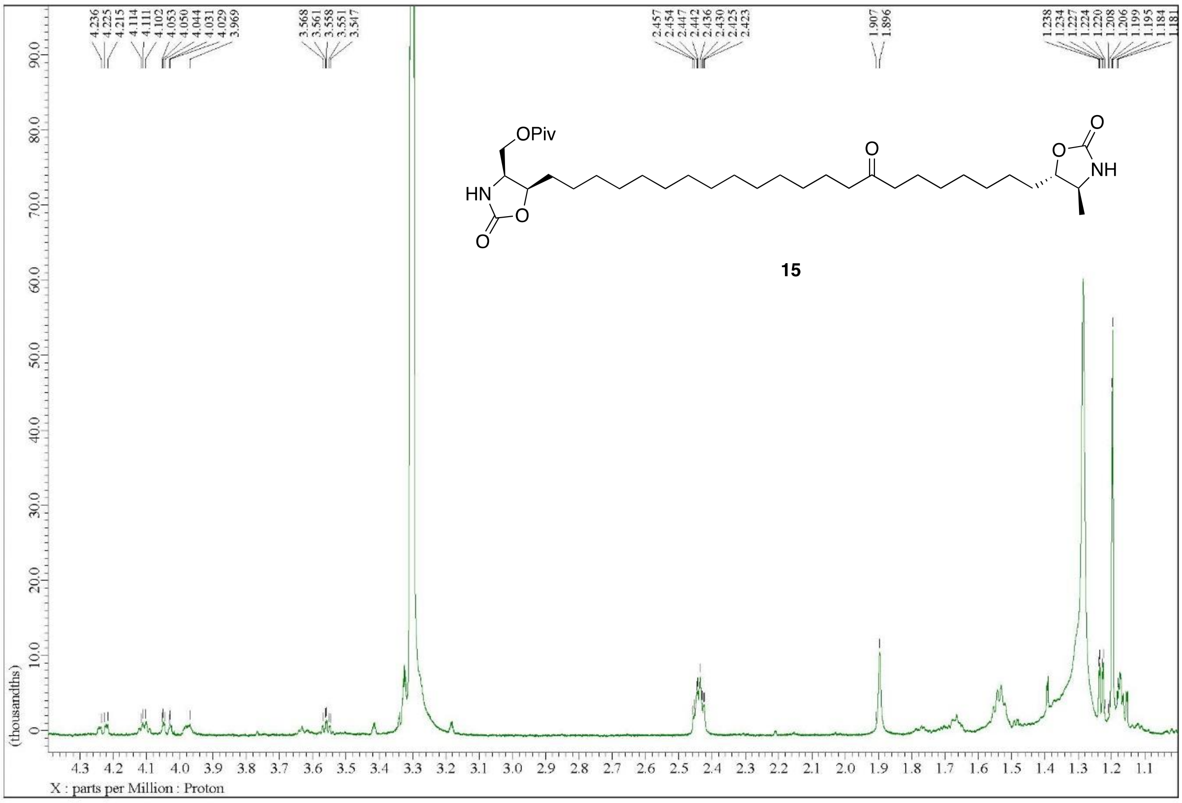

Figure S21. ^{1}H NMR spectrum (600 MHz) of the bis-oxazolidinone (**15**) in CD_3OD

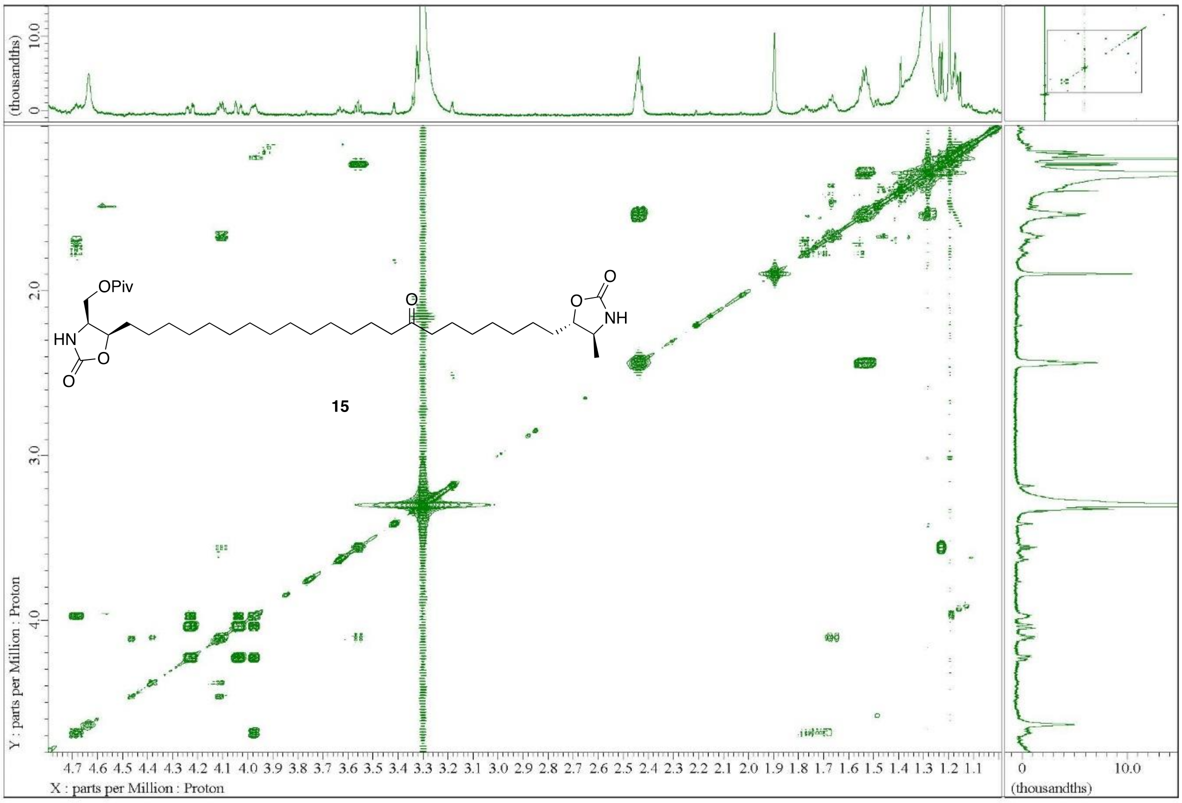

Figure S22. COSY spectrum (600 MHz) of the bis-oxazolidinone (**15**) in CD_3OD

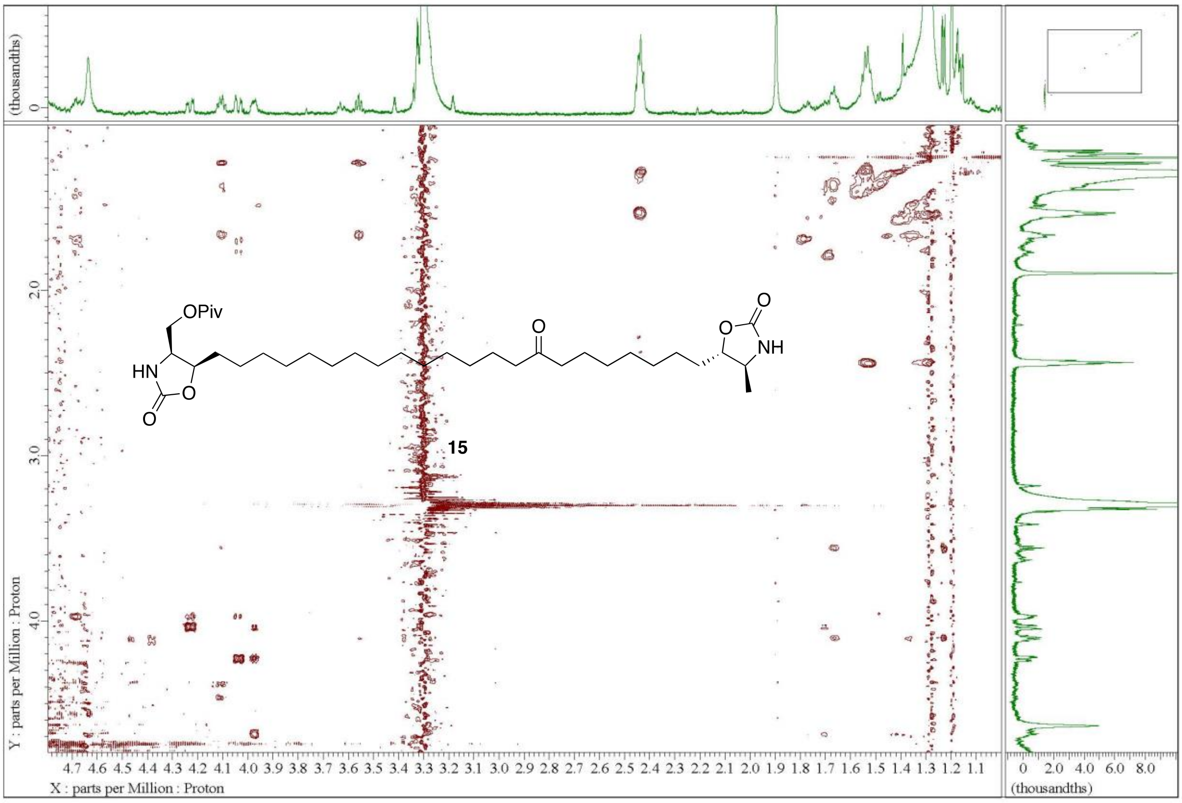

Figure S23. NOESY spectrum (600 MHz) of the bis-oxazolidinone (**15**) in CD_3OD

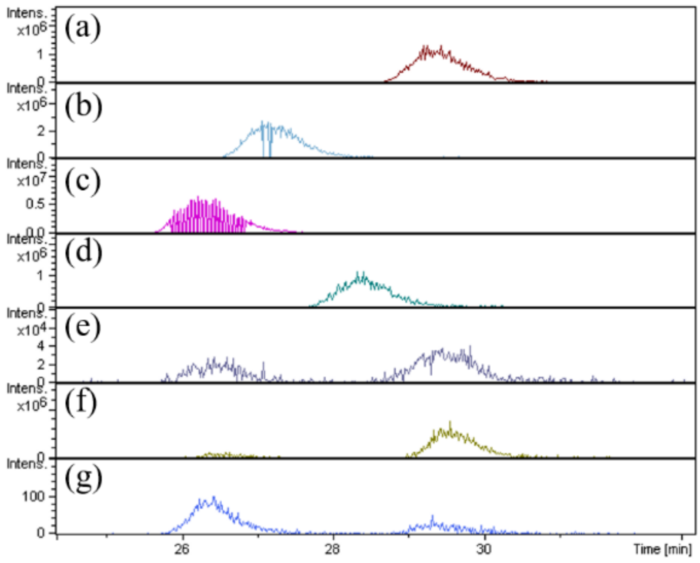

Figure S24. LC–MS chromatograms of sugar derivatives

a: D-glucose, b: L-glucose, c: D-galactose, d: L-galactose, e: hydrolysate of **1** and **2**, f: D-glucose and hydrolysate of **1** and **2**, g: D-galactose and hydrolysate of **1** and **2**

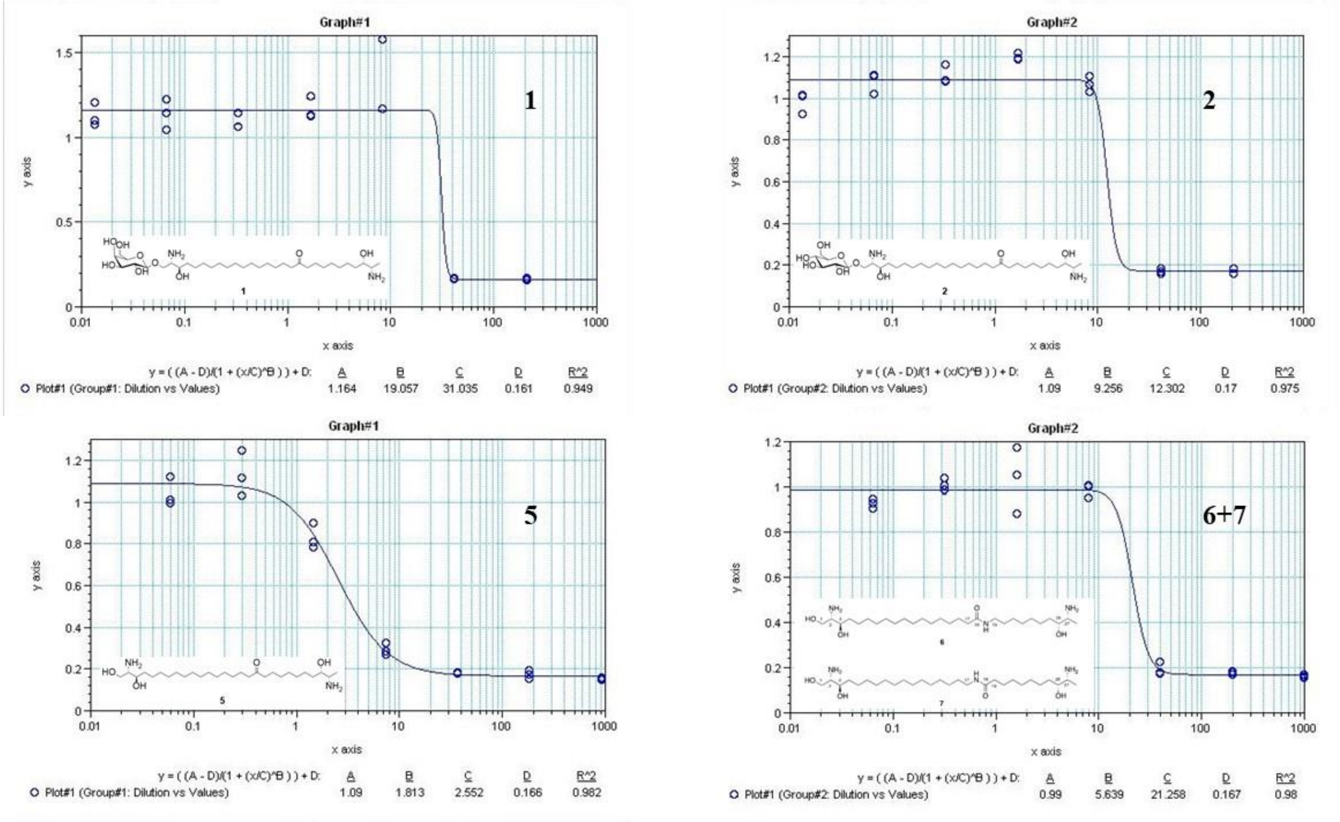

Figure S25. Graphs of MTT assay of **1**, **2**, **5** and the mixture of **6** and **7** against HeLa cells
